# Engineering a cell-based orthogonal ubiquitin transfer cascade for profiling the substrates of RBR E3 Parkin

**DOI:** 10.1016/j.isci.2025.112913

**Published:** 2025-06-17

**Authors:** Shuai Fang, Li Zhou, Geng Chen, Jing Zhang, Xiaoyu Wang, In Ho Jeong, Savannah E. Jacobs, Bradley R. Kossmann, Wei Wei, Shu Liu, Geon H. Jeong, Yayun Xie, Duc Duong, Nicholas T. Seyfried, Ivaylo Ivanov, Angela M. Mabb, Hiroaki Kiyokawa, Bo Zhao, Jun Yin

**Affiliations:** 1 Engineering Research Center of Cell and Therapeutic Antibody, Ministry of Education, and School of Pharmacy, Shanghai Jiao Tong University, Shanghai, China; 2 Department of Chemistry and Center for Diagnostics and Therapeutics, Georgia State University, Atlanta, GA 30303, USA; 3 Neuroscience Institute and Center for Behavioral Neuroscience, Georgia State University, Atlanta, GA 30302, USA; 4 Depatrment of Biochemistry, Emory University School of Medicine, Atlanta, GA 30322, USA; 5 Department of Pharmacology, Northwestern University, Chicago, IL 60611, USA

**Keywords:** Biochemistry, Protein, Biomolecular engineering, Structural biology, Methodology in biological sciences

## Abstract

The E3 ubiquitin (UB) ligase Parkin utilizes a Ring-Between-Ring (RBR) domain to mediate UB transfer to substrate proteins, and mutations affecting Parkin catalysis promote cancer and are associated with Parkinson’s disease. An essential role of Parkin is to initiate mitophagy by ubiquitinating mitochondrial proteins. Still, it is unclear how Parkin carries out other cellular functions, such as the regulation of the cell cycle, metabolism, and the neuronal synapse. Here, we used phage display to engineer the RBR domain of Parkin and assembled an orthogonal ubiquitin transfer (OUT) cascade to profile Parkin substrates in living cells. Guided by the substrate profile from the OUT screen, we verified a panel of Rab GTPases and CDK5 as Parkin substrates. We also showed mitophagy stimulation enhanced Parkin-mediated ubiquitination of Rab proteins. Our work demonstrates that the OUT cascade can be an empowering tool for identifying Parkin substrates to elucidate its multifaceted cellular functions.

## Highlights

• Redesign of the Parkin RBR-ubiquitin interface by modeling and phage display
• Parkin substrate profile in HEK293 cells revealed by orthogonal ubiquitin transfer
• Parkin catalyzes the ubiquitination of Rab GTPases and CDK5
• Mitochondria damage stimulates Rab ubiquitination by Parkin

## Graphical abstract

**Figure:**
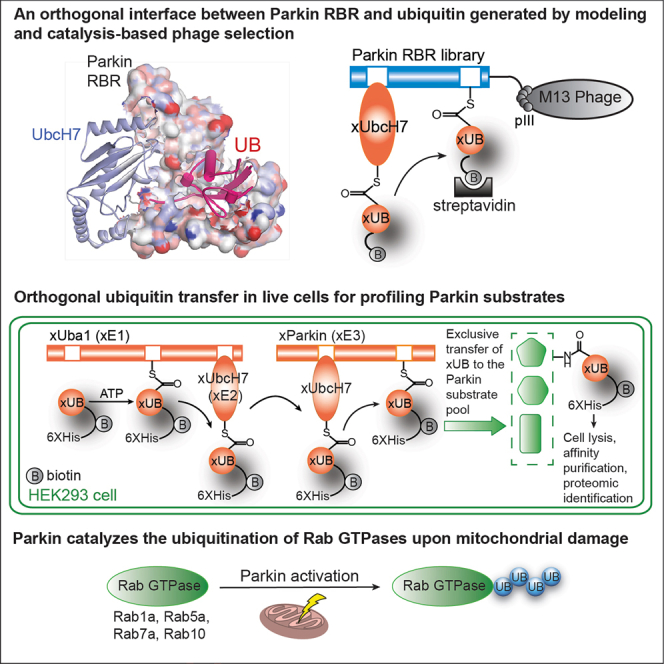

## Introduction

Parkin is a Ring-Between-Ring (RBR) type of E3 ubiquitin (UB) ligase that regulates diverse cellular processes, including mitophagy, energy metabolism, cell cycle progression, and synaptic activity.^1^^,^^2^^,^^3^^,^^4^^,^^5^^,^^6^ The best-characterized role of Parkin is its engagement in a feedforward cycle with the PTEN-Induced Kinase 1 (PINK1) to ubiquitinate proteins on damaged mitochondria for mitophagy initiation.^7^^,^^8^^,^^9^ In such a cycle, PINK1 is stabilized on the outer mitochondrial membrane (OMM) upon mitochondria depolarization and phosphorylates UB conjugated to OMM proteins. Phosphorylated UB molecules then bind to Parkin and recruit it to the mitochondrial surface for PINK1-catalyzed phosphorylation that would release Parkin from an auto-inhibited state.^10^^,^^11^^,^^12^^,^^13^ Activated Parkin synthesizes more UB chains on the mitochondrial surface for their phosphorylation by PINK1, which in turn recruits and activates more Parkin. Loss-of-function mutations in Parkin and PINK1 are the first and second most common causes of autosomal recessive Parkinson’s disease (PD), suggesting that defects in the mitochondria stress response may impinge on neurodegenerative processes associated with PD.^14^^,^^15^ However, endogenous Parkin in neuronal cells was not able to induce mitophagy upon chemical damage of mitochondria, and the PINK1-Parkin pathway did not affect basal mitophagy in mice and Drosophila.^16^^,^^17^^,^^18^ These findings suggest Parkin may regulate other cellular processes underpinning PD pathogenesis independent of mitophagy, and a profile of Parkin substrates would be instrumental in deciphering these unexplored roles of Parkin in PD.

Parkin does not demonstrate strict substrate specificity in ubiquitinating OMM proteins for building UB chains on damaged mitochondria.^2^^,^^19^ Still, the choice of early ubiquitination targets by Parkin on the OMM is important for mitophagy initiation. For example, Parkin ubiquitinates mitofusin 1 and 2 (MFN1/2), two transmembrane GTPases on the OMM, to signal their degradation by the proteasome.^20^^,^^21^ The removal of MFN1/2 stops mitochondria fusion, so damaged mitochondria do not contaminate their healthy counterparts. Parkin also ubiquitinates Rho family GTPases Miro1 and Miro2 on the OMM, and their induced degradation freezes the movement of damaged mitochondria on the cytoskeleton to prepare them for mitophagy.^22^^,^^23^ Besides mitophagy, the PINK1-Parkin axis may also operate on cytosolic targets, such as PARIS, the transcriptional co-repressor, and affect mitochondrial biogenesis. Parkin-mediated ubiquitination and degradation of PARIS enhances the expression of PGC-1α to promote mitochondrial biogenesis, which has a protective effect on dopamine-producing neurons.^24^^,^^25^^,^^26^

Parkin can also be activated in the cytosol by binding to phosphorylated UB generated by PINK1 or through the oxidation of its Cys residues.^27^^,^^28^ Furthermore, kinases other than PINK1, such as AMP-activated protein kinase (AMPK), Unc-51-like autophagy activating kinase 1 (ULK1), and Ca^2+^/calmodulin-dependent protein kinase 2 (CaMK2), have been found to phosphorylate and activate Parkin to regulate mitophagy, necroptosis, and synaptic vesicle recycling.^29^^,^^30^^,^^31^ The non-mitochondrial function of Parkin plays a key role in tumor suppression by ubiquitinating proteins involved in cell proliferation, apoptosis, and metabolism.^32^ For example, Parkin cooperates with CDC20 and CDH1, the subunits of a cell cycle-regulatory E3 known as the anaphase-promoting complex/cyclosome (APC/C), to mediate the ubiquitination and degradation of mitotic regulators, such as Polo-like kinase-1, Aurora-A/B, and Cyclin B1, exerting anti-proliferative effects.^33^ Parkin, by itself, can ubiquitinate cyclin E and D1 to regulate their stability and the cell cycle.^34^^,^^35^ It also ubiquitinates and inhibits pyruvate kinase M2 (PKM2) without inducing its degradation, which further suppresses the Warburg effect that is crucial for cancer cell metabolism.^36^ These tumor-suppressive actions of Parkin are consistent with the genetic and epigenetic inactivation of the *PRKN* gene found in a variety of human cancers.^32^^,^^37^ In the apoptotic pathways, Parkin can induce cell death by ubiquitinating and degrading Mcl1, an anti-apoptotic protein of the Bcl2 family.^38^^,^^39^ On the other hand, Parkin also ubiquitinates pro-apoptotic proteins BAK and BAX, which inhibits their oligomerization or translocation to the mitochondria to prevent mitochondria membrane permeabilization and the subsequent release of cytochrome *c* as an apoptotic signal.^40^^,^^41^ This allows Parkin to maintain mitochondria homeostasis and promote cell survival. Besides PD and cancer, Parkin has been implicated in multiple diseases, including leprosy, cerebral ischemia, and autism spectrum disorder.^42^^,^^43^^,^^44^ Developing unbiased methods to profile Parkin substrates will facilitate the deconvolution of the pathogenic roles of Parkin.

Parkin, like other E3s, carries out the last step of UB transfer through the E1-E2-E3 enzymatic cascades to conjugate UB to its substrate proteins. There are more than 600 E3s encoded in the human genome,^45^^,^^46^ including 14 RBR types of E3s,^47^ and their combination with 2 E1s and ∼40 E2s^48^^,^^49^^,^^50^ constitute a complex network of UB transfer cascades and make it a significant challenge to identify the direct substrates of an E3 for interpreting its biological functions. To identify the substrates of a designated E3 in the cell, we constructed orthogonal UB transfer (OUT) cascades of E3s consisting of engineered xE1-xE2-xE3 enzymes to exclusively transfer a UB mutant (xUB) through a designated xE3 to its ubiquitination targets for their identification by proteomics.^51^ We have validated the OUT platform for profiling E3 substrates by constructing OUT cascades with HECT type E3s E6AP/UBE3A and Rsp5, and U-Box E3s CHIP and UBE4B, and identified their substrates important for cell metabolism, antiviral response, protein aggregation, and endoplasmic reticulum (ER)-associated stress.^52^^,^^53^^,^^54^^,^^55^ In this study, we engineered the RBR domain of Parkin to extend the OUT cascade to RBR E3s for profiling their cellular targets. Based on the substrate profile generated by OUT, we found potential roles of Parkin regulating membrane vesicle trafficking by verifying Parkin-catalyzed ubiquitination of a panel of Rab GTPases, including Rab1a, Rab5a, Rab5c, Rab7a, Rab8a, Rab10, and Rab13, that support vesicle trafficking in the cell. We also verified Parkin catalyzed ubiquitination of cyclin-dependent kinase 5 (CDK5), whose hyperactivation was implicated in neurodevelopment and neurodegenerative processes. We found Parkin reduced the levels of Rab and CDK5 proteins in the cell, and enhanced Parkin activity coupled with mitophagy induction stimulated the ubiquitination of Rab proteins. Currently, several studies have been reported in the literature on identifying Parkin substrates by following the change of protein ubiquitination levels with the enrichment of diGly-modified peptide or purification of ubiquitinated proteins by tandem UB-binding entities (TUBEs).^9^^,^^19^^,^^56^^,^^57^^,^^58^^,^^59^ The OUT cascade identified Parkin substrates by directly following xUB transfer from Parkin to its substrate proteins. It overcomes the cross-regulation between Parkin and other E3s in the cell that may indirectly affect the ubiquitination levels of the substrate proteins of other E3s. The construction and validation of the Parkin OUT cascade in this work provide an empowering tool for elucidating diverse Parkin functions in normal cell physiology and disease development.

## Results

### Design and construction of RBR libraries of Parkin for engineering the OUT cascade

**Figure 1:**
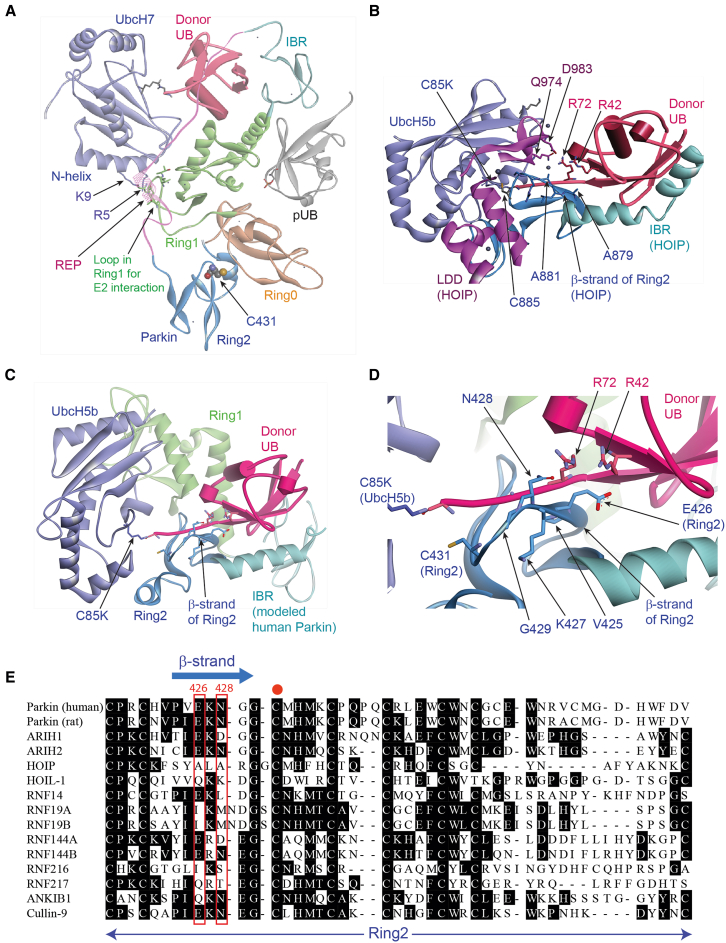
Structure analysis of the binding interface between the Parkin RBR domain and the UB ∼ UbcH7 conjugate for engineering the OUT cascade with Parkin (A) The structure of human Parkin binding with the UbcH7∼UB conjugate (PDB ID: 6N13).^62^ The structure was derived from NMR chemical shift perturbation experiments, and it shows the binding between the N-terminal helix of UbcH7 and the loop region of the Ring1 domain of Parkin. (B) Crystal structure of HOIP RBR bound with the UbcH5b ∼ UB conjugate showing the UB C-terminus in an extended conformation and making contacts with the residues in the Ring2 and LDD domain of HOIP (PDB ID: 5EDV).^64^ (C) Modeled structure of human Parkin RBR in a complex with the UbcH5b ∼ UB conjugate from this study. The model was generated to mimic the structure complex between the RBR domain of HOIP and the UbcH5b ∼ UB conjugate in (B). (D) A detailed view of the interaction between the RBR domain of human Parkin with the donor UB bound to UbcH5b as shown in the modeled structure in (C). R42 and R72 of UB are in close vicinity to the residues constituting the β-strand with the sequence ^425^VEKNG^429^. (E) The alignment of the protein sequences of the Ring2 domain of the RBR E3s, including human and rat Parkin and HOIP. Residues 426 and 428 (numbering in human Parkin) in the β-strand of Ring2 for interacting with R42 and R72 of UB were highlighted in red frames. The catalytic Cys residue of the Ring2 domain is marked by a red dot.

The OUT cascade of Parkin was designed to exclusively transfer an engineered xUB with the R42E and R72E mutations to Parkin and then to its substrate proteins in the cell. The double mutations in xUB prevent its activation by a wildtype (wt) E1 (Uba1 or Uba6). Instead, xUB would be activated by xE1 (xUba1) with an engineered UB-binding site and E2-binding site for engaging an engineered xE2 (xUbcH5 or xUbcH7) to transfer xUB from the xE1 to xE2.^60^ xUbcH7 has the R5E and K9E mutations in its N-terminal helix that would match with the redesigned E2-binding site in the UB-fold domain (UFD) of xUba1.^60^ We found xUB cannot be transferred from the xUba1-xUbcH7 pair to wt Parkin, suggesting the mutated interfaces in the xUB ∼ xUbcH7 conjugate are incompatible with endogenous Parkin to mediate UB transfer (Figure S1). The mutated residues in xUB and xUbcH7 are at different interfaces with Parkin. The residues in the N-terminal helix of UbcH7 would interact with a loop in the Ring1 domain of Parkin as shown by a structure of human Parkin bound to the UbcH7∼UB complex solved by NMR measurements and a crystal structure of *B. dorsalis* (oriental fruit fly) Parkin in a complex with UbcH7 (Figures 1A and S2A).^61^^,^^62^ The equivalent sequence in the Ring1 domain of rat Parkin for interacting with the N-terminal helix of UbcH7 would be ^237^PCIACTD^243^ with residues P237, I239, A240, T242, and D243 constituting the binding interface, as shown by the crystal structure of rat Parkin (Figures S2 and S2B–S2D).^63^ We thus randomized these residues in the RBR domain of rat Parkin for selecting mutants with restored activity with the xUB-xUbcH7 conjugate to mediate xUB transfer.

The crystal structure of the RBR domain of HOIL-1-Interacting Protein (HOIP) with the UB ∼ UbcH5b conjugate revealed a binding interface between the R42 and R72 residues of UB and the Ring2 and LDD domain of HOIP (Figure 1B).^64^ In the complex structure, the UB ∼ UbcH5b conjugate adopts an “open” conformation due to the tight engagement of Ring2 and LDD with the UB C-terminal residues ^71^LRLRGG^76^ that connect the catalytic Cys residue of UbcH5b with the globular domain of UB. The interactions between HOIP RBR and the UbcH5b ∼ UB conjugate also position the catalytic Cys (C885) in the Ring2 domain of the RBR within attacking distance from the thioester bond of the UbcH7∼UB conjugate to enable the transthiolation reaction. The complex structure also showed that the R42 and R72 residues of UB, which are mutated to Glu in xUB, may engage LDD residues Q974 and D983 of HOIP through salt bridge and hydrogen bonding interactions. In addition, R42 and R72 of UB may interact with the β-sheet residues (^87^^^8^^YALARG^883^) in the Ring2 domain of HOIP that runs in parallel to the C-terminal tail of UB (Figure 1B). Parkin does not have an LDD domain equivalent to HOIP, but the corresponding β-sheet residues in the Ring2 domain of rat Parkin have the sequence of ^425^IEKNG^429^ with residues E426 and N428 likely interacting with R42 and R72 of UB in the UB ∼ UbcH7 conjugate. To reveal the interaction between the Ring2 domain of Parkin and the UB ∼ E2 conjugate, we modeled the structure of the human Parkin RBR domain after the crystal structure of HOIP RBR with both RBRs bound with the UB ∼ UbcH5b conjugate (Figure 1C). In the modeled structure of the Parkin RBR, the β-strand containing the sequence ^425^VEKNG^429^ in the Ring2 domain of human Parkin is indeed in contacting distance to R42 and R72 of UB, and E426 and N428 within the Ring2 β-strand may engage the two Arg residues of UB with electrostatic and hydrogen bonding interactions (Figures 1D and 1E). The newly reported NMR structure of a Ring2 fragment of the *D. melanogaster* Parkin with a donor UB conjugated to the catalytic Cys residue showed similar interactions between R42 and R72 of UB with the corresponding residues in the Ring2 domain of Parkin (Figure S3A).^65^ The structure model of human full-length Parkin undergoing the transthiolation reaction with the UbcH7∼UB conjugate was reported in the same study, and it matches our modeling of the RBR interaction with UB bound to E2 (Figure S3B).^65^ We thus decided to construct a second rat Parkin library with randomized residues replacing I425, E426, N428, and G429 to restore the interaction between the RBR domain and xUB and enable xUB transfer through the engineered xParkin.

### Development of a phage display system for catalysis-based selection of the RBR domain of Parkin

**Figure 2:**
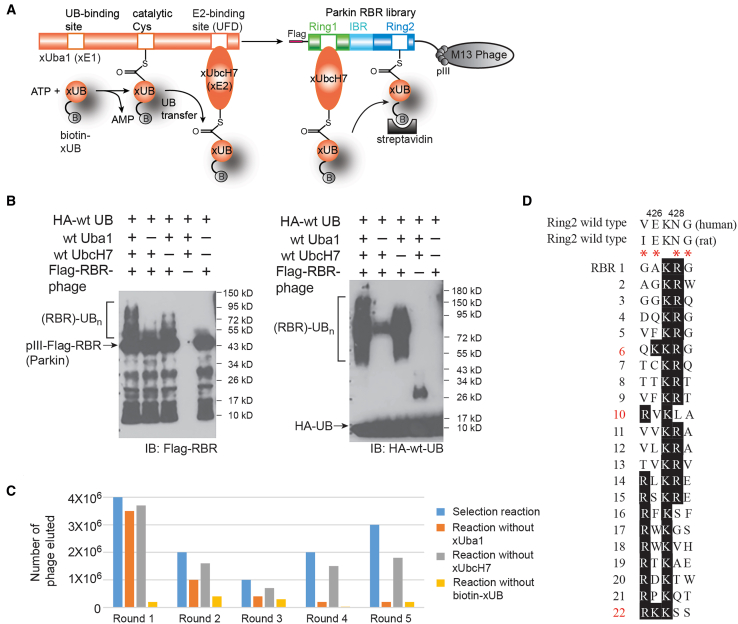
Phage selection of the RBR library of Parkin (A) Selection of the RBR library of Parkin was based on the catalytic transfer of biotin-labeled xUB from xUba1 and xUbcH7 to the RBR variants displayed on the M13 phage. Once xUB was attached to the RBR domain on the phage surface with the formation of the thioester conjugate, the corresponding phage particles were labeled by biotin and affinity selected by the interaction between biotin and immobilized streptavidin. (B) Reactivity of wt RBR domain of Parkin displayed on the phage particle. Ubiquitination reactions of the RBR domain on the surface of M13 phage were set up with HA-tagged wt UB (HA-wt UB), Uba1, UbcH7, and phage displaying the RBR domain. In control reactions, Uba1, UbcH7 or RBR displayed phage were excluded to establish the dependence of UB transfer to RBR through the cascade enzymes. The reactions were treated with a gel-loading dye containing 50 mM DTT before SDS-PAGE analysis and western blotting. (C) Selection of the RBR library of rat Parkin with randomized residues in the β-strand of the Ring2 domain. The phage library underwent five rounds of selection until clones from the selection showed convergent sequences at the randomized sites. In control reactions, xUba1, xUbcH7 or biotin-xUB was excluded from the reaction. Eluted phage titers from the selection reaction were greater than the control reactions, demonstrating the dependence of selection on the catalytic transfer of xUB from the xUba1-xUbcH7 pair to the RBR domain displayed on phage. (D) Alignment of the sequences of the RBR clones from the 5th round of selection of the library, showing convergence of the selected clones in the randomized β-strand region of Ring2. The randomized residues in Ring2 are designated by red stars.

We developed a phage display system for the selection of the RBR domain of Parkin based on its catalytic activity in pairing with xUbcH7 for the transfer of xUB (Figure 2A). In this system, the RBR domain library was expressed and anchored on the phage surface as an N-terminal fusion to the phage capsid protein pIII. The reaction of the RBR library with the xUba1-xUbcH7 pair would enable the transfer of biotin-xUB to the phage-displayed RBR if the RBR variants had acquired complementary mutations to restore their interaction with the xUbcH7∼xUB conjugate. The formation of the RBR ∼ xUB conjugate would lead to biotinylation of the corresponding phage particles, and they could be selected by binding to plates immobilized with streptavidin. Phage displaying catalytic active RBR domains were eluted by reaction with dithiothreitol (DTT) to cleave the thioester linkage between biotin-xUB and RBR. The eluted phage was then amplified by infecting *E. coli* cells for a subsequent round of selection. Through several rounds of phage selection, we expected to see the randomized residues in the RBR library to converge to a few consensus sequences to reveal the identity of catalytically active RBR variants that could assemble with the xUba1-xUbcH7 pair for xUB transfer. The RBR ∼ xUB thioester conjugate formed on the phage surface would be transient and unstable, and xUB in the conjugate could be further transferred to Lys residues on the RBR to generate self-ubiquitinated products. We thus expected only a small fraction of the RBR-displayed phage would yield the RBR ∼ xUB thioester conjugate that could be released from streptavidin by DTT cleavage. Still, the large number of phage particles in the selection reaction (∼10^11^) might play to our advantage to overcome this issue.

To validate the phage selection strategy, we displayed the wt RBR domain of Parkin on the M13 phage and incubated the phage with wt Uba1, wt UbcH7, and HA-wt UB to check the loading of UB on the RBR domain. In the presence of wt Uba1 and UbcH7, we observed strong ubiquitination of the RBR-pIII fusion displayed on phage, and the UB loading on the RBR was dependent on Uba1 and UbcH7 (Figure 2B). In the absence of the E2 enzyme UbcH7, RBR could still be loaded with UB, but to a lesser extent, which was expected since the E2-independent activity of RBR has been shown to support self-ubiquitination of RBR in reconstituted reactions.^66^ We also compared the formation of RBR-UB conjugates on the phage surface before and after treatment of DTT and found no significant accumulation of the RBR ∼ UB thioester conjugate in the self-ubiquitination reaction without DTT treatment (Figure S4A). This indicates that the RBR ∼ UB thioester conjugate, if formed, would be much less than the polyubiquitinated RBR species observed on the western blot. We thus relied on the phage model selection to validate the selection strategy for catalytically active RBR. We first carried out model selections for wt RBR displayed phage in parallel with a control phage that displayed an SV5V viral protein.^67^ Both types of phage were reacted with wt Uba1 and UbcH7 to transfer biotin-wt-UB to the RBR. After the reaction, the phage was bound to the streptavidin plate and eluted with DTT. The phage titer showed that, with the same amount of phage input, the number of RBR phage retained by the streptavidin plate was more than 100-fold higher than that of the SV5V phage, demonstrating the high efficiency for selecting the RBR displayed phage based on the catalytic transfer of biotin-wt-UB through the Uba1-UbcH7 pair (Figure S4B). We then carried out model selection for Parkin RBR displayed phage from mixtures with SV5V displayed phage. After the reaction of the phage mixtures with biotin-wt UB and the Uba1-UbcH7 pair, the phage mixtures were selected by binding to the streptavidin plate, and the eluted phage particles were used to infect *E. coli* cells. We then used colony PCR to differentiate colonies infected with RBR or SV5V-displayed phage and found 8 out 10 clones selected from the 1/100 mixture of RBR/SV5V phage contained the pComb-RBR plasmid, and 1 out of 10 clones selected from the 1/1,000 mixture of RBR/SV5V phage contained the pComb-RBR plasmid (Figure S4C). Therefore, each round of phage selection was able to enrich Parkin RBR phage by almost 100-fold, confirming the high efficiency of the phage display for engineering RBR variants based on UB transfer.

### Engineering the RBR domain of Parkin by phage display

We first carried out selection with the rat RBR library of Parkin with randomized loop residues in Ring1 that would interact with the N-terminal helix of UbcH7 (Figures 1A and S2). We reacted the phage library with biotin-xUB and the xUba1-xUbcH7 pair so biotin-xUB could be transferred to the RBR variants on phage if there were restored interactions. However, after five rounds of selection, we could not achieve convergence in the Ring1 library. These findings suggested that other interfaces, such as that between the R42E and R72E mutations in xUB and the Ring2 domain of RBR, could be important for restoring the interaction of RBR with the xUB ∼ xUbcH7 conjugate. We thus carried out phage selection with the rat RBR library randomized in the β-strand of Ring2 (Figure 1C). After five rounds of selection with increasing stringency, we observed the enrichment of phage particles from the selection reaction with the xUba1-xUbcH7 pair and biotin-xUB over controls (Figure 2C). In the fifth round of selection, we observed a 20- to 30-fold higher number of phage particles eluted from the selection reaction compared to the controls, suggesting the phage selection was dependent on the catalytic transfer of biotin-xUB from the xUba1-xUbcH7 pair to the catalytic active RBR variants displayed on phage.

**Figure 3:**
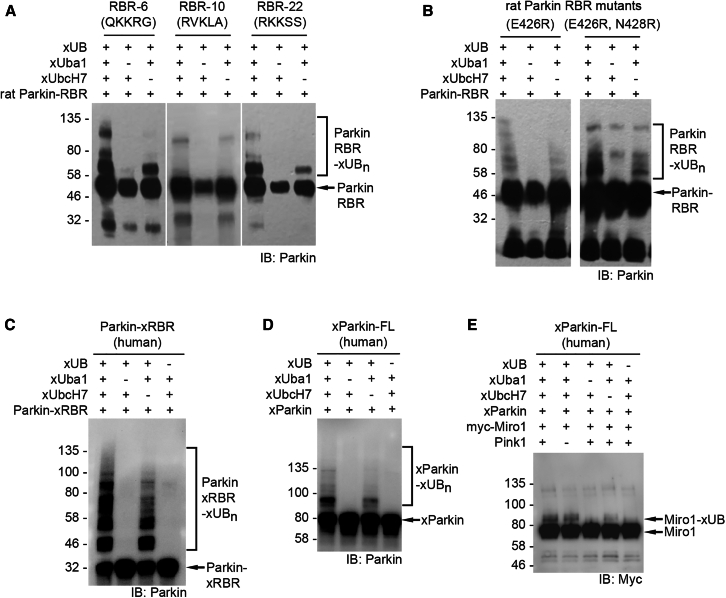
The xUB transfer activity of RBR variants from the selection of phage library with randomized Ring2 residues (A) xUB transfer catalyzed by clones 6, 10 and 22 of the RBR mutants in the self-ubiquitination reaction. Clone 6 demonstrated the highest activity in xUB transfer. The RBR mutants were expressed as GST fusions. (B) Self ubiquitination activity of rat Parkin RBR with single mutation E426R and double mutation E426R/N428R. The RBR mutants were expressed as GST fusions. (C) The RBR domain of human Parkin with the E426R/N428R double mutations could mediate xUB transfer in the self-ubiquitination reaction. (D) The full-length (FL) human Parkin with the E426R/N428R double mutations also underwent self-ubiquitination with xUB. The Parkin mutant was expressed as a GST fusion. (E) Engineered human Parkin with the E426R/N428R double mutations could transfer xUB to known Parkin substrate Miro1.

Sequencing of the rat Parkin RBR variants from the fifth round of selection revealed a strong preference for N428 to be mutated to a positively charged Arg or Lys residue within the sequence of ^425^IEKNG^429^ that was randomized in the Ring2 library (Figure 2D). In addition, the negatively charged residue Glu426 in the wt Parkin RBR was mutated to hydrophobic residues such as Val or Trp or positively charged residues like Lys or Arg. We picked several representative clones to screen their activity in mediating xUB transfer from the xUba1-xUbcH7 pair (Figure 3A). The selected rat RBR domains were expressed as GST fusions, and the self-ubiquitination assay showed clone 6 with the sequence ^425^QKKRG^429^ and clone 22 with the sequence ^425^RKKSS^429^ had the highest activity in self-ubiquitination through xUB transfer from xUba1-xUbcH7. In contrast, clone 10 with the sequence ^425^RVKLA^429^ did not show strong activity of xUB transfer, suggesting positively charged Lys or Arg residues in position 426 or 428 in the Ring2 domain of the RBR were crucial for restoring its interaction with xUB. Based on the sequence alignment of the selected clones, there was not a significant consensus for the residues at position 425, which corresponded to an Ile in rat Parkin or a Val in human Parkin, and at position 429 with a Gly in the native rat and human sequences (Figures 1E and 2D). We thus kept the native residues of I425 and G429 and incorporated the double mutations of E426R and N428R into the rat Parkin RBR domain. Generation of these mutations restored the self-ubiquitination of the engineered xRBR with xUB, suggesting the mutations could restore the binding of xUB in the xUbcH7∼xUB conjugate with the RBR domain (Figure 3B). In contrast, rat Parkin RBR with the single E426R mutation was less active with xUB transfer, suggesting both E426R and N428R mutations were needed to complement the R42E and R72E mutations in the xUB to enable the transfer of xUB to the xRBR domain. We next incorporated the E426R and E428R mutations into the RBR domain of human Parkin and found the mutant RBR could undergo efficient self-ubiquitination by up-taking xUB from the xUba1-xUbcH7 pair (Figure 3C). The full-length human Parkin with the same set of mutations in the RBR could also transfer xUB in the self-ubiquitination reaction (Figure 3D). Furthermore, human Parkin with the E426R and N428R mutations could transfer xUB to Miro1, a well-characterized Parkin substrate (Figure 3E).^68^ Cumulatively, our results show that the Parkin mutant we engineered by phage display can function as an xParkin for the assembly of the OUT cascade to transfer xUB to Parkin substrates.

### Profiling the substrates of Parkin with the OUT cascade

**Figure 4:**
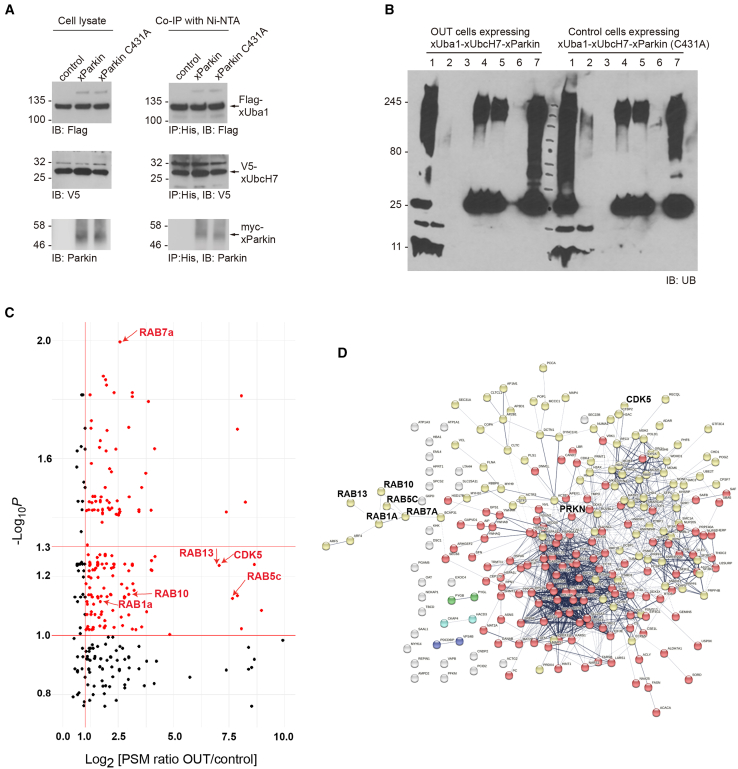
Expression of the OUT cascade of Parkin in HEK293 cells and identification of xUB-conjugated proteins (A) Expression of the Parkin OUT cascade in HEK293 cells and confirmation of the activity of the OUT cascade in the transfer of xUB by assaying the presence of the OUT enzymes in the Ni-NTA pulldown samples. (B) Tandem purification of xUB-conjugated proteins from lysates of cells expressing the catalytically active OUT cascade (OUT cells) and the control cascade with the C431A mutant of xParkin (control cells). Lane assignments: 1. cell lysates before Ni-NTA binding; 2. flow-through of cell lysates after Ni-NTA binding; 3. wash of Ni-NTA beads; 4. elution from Ni-NTA beads; 5. flow-through from streptavidin beads; 6. wash of streptavidin beads; 7. proteins bound to streptavidin beads. The western blot was probed with an anti-UB antibody to reveal ubiquitinated species enriched by tandem purification. (C) Volcano plot of Parkin substrates identified by the OUT screen. *N* = 3 independent biological replicates. Red dots designate proteins with Log_2_[PSM ratio OUT/control] >1 and -Log_10_*p* > 1. This plot amplified the region containing Rab proteins and CDK5 that were verified as Parkin substrates in this study. The full volcano plot is shown in Figure S5B. (D) Protein-protein interaction network for the targets identified from the OUT screen of Parkin substrates. The plot was generated from STRING (STRING PPI enrichment *p*-value <1.0e-16). Line thickness indicates the strength of data support.

The construction of the OUT cascade of Parkin gave us a platform for identifying Parkin substrates in cells. We previously screened HEK293 cells that would stably express the xUba1-xUbcH7 pair.^52^ We co-expressed plasmids of myc-xParkin and xUB in HEK293 cells stably expressing the xUba1-xUbcH7 pair for the assembly of the OUT-cascade in the cell. xUB contains an N-terminal 6×His and BCCP (biotin carboxyl carrier protein) tag (HBT-xUB) for biotin tagging. The N-terminal myc tag fused to Parkin may perturb its auto-inhibited state, leading to an increase in its activity without PINK1 activation.^69^ To assay the expression of OUT components, we probed the expression of OUT enzymes in the cell via western blotting using antibodies against the specific tags fused to xUba1 (Flag), xUbcH7 (V5), and xParkin (myc) (Figure 4A). To confirm the activity of the OUT cascade in transferring xUB in the cell, we purified HBT-xUB conjugates from cell lysates using Ni-NTA resin and verified the presence of xUba1, xUbcH7, and xParkin in the resin-bound fraction (Figure 4A). These results suggested the OUT cascade could be expressed in HEK293 cells and was active in transferring xUB to xParkin. As a control, we performed a mock purification of the OUT components in cells lacking HBT-xUB and found none of xUba1, xUbcH7, or xParkin was present in the fraction bound to the Ni-NTA resin (Figure S5A). We also generated the C431A mutant of xParkin with its catalytic Cys residue mutated to Ala and expressed the C431A mutant with the xUba1-xUbcH7 pair and HBT-xUB in HEK293 cells (Figures 4A and S5A). The C431 mutant was expressed at a similar level as the catalytic active xParkin in the cell, and its presence in the fraction of HBT-xUB conjugated proteins bound to the Ni-NTA resin was slightly decreased, suggesting it could be modified by xUB due to the association of xParkin-C431A with the xUbcH7-xUB conjugate. Since the C431 mutation is known to inhibit the transfer of UB to the Parkin substrates,^70^ we used cells expressing the xUba1-xUbcH7 pair and xParkin-C431A as control cells to identify proteins that were preferentially labeled with HBT-xUB in cells expressing the catalytically active OUT cascade (OUT cells). In parallel runs, we purified xUB-conjugated proteins from the cell lysate of OUT cells and control cells using Ni-NTA and streptavidin affinity columns under denaturing conditions and digested the proteins bound to the streptavidin resin with trypsin for proteomic analysis (Figure 4B).

**Figure 5:**
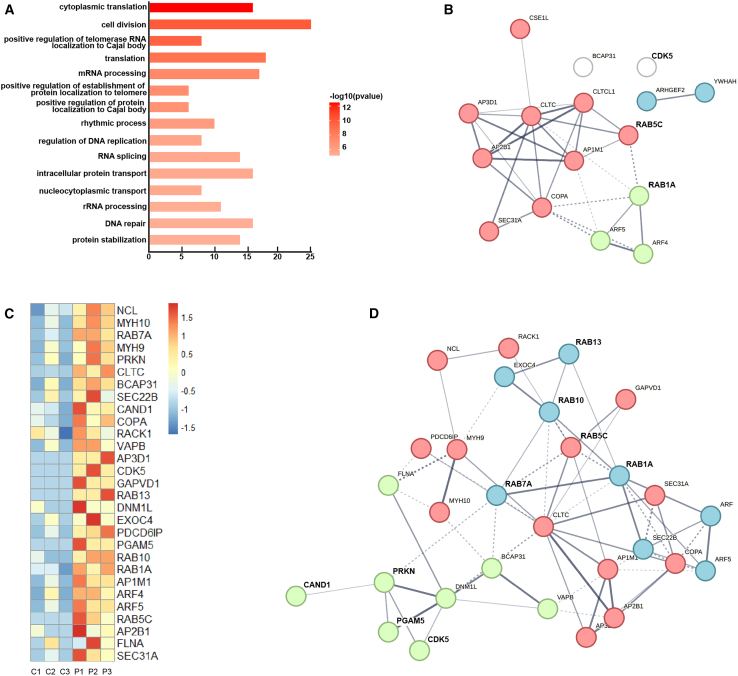
Analysis of the biological functions and interactions of Parkin substrates identified by the OUT screen (A) Visualization of top significant terms from the list of gene ontology related to Biological Process. (B) STRING protein-protein interaction network for the targets identified from OUT that were classified under intracellular protein transport (GO:006886). (C) Heatmap with normalized PSM values for the targets identified from OUT that were categorized under vesicle-mediated transport (GO:0016192). P1-P3: 3 replicates from OUT cells expressing the functional OUT cascade of Parkin; C1-C3: 3 replicates from the control cells with the C431 mutation in xParkin. (D) STRING protein-protein interaction network for targets identified from OUT that were classified under vesicle-mediated transport.

We repeated the tandem purification and proteomic identification of xUB-conjugated proteins three times from Parkin OUT and control cells cultured in parallel to acquire the proteomic datasets for the identification of Parkin substrates (Table S1). The volcano plot of the dataset identified 254 potential Parkin substrates with a *p* value <0.1 (-Log_10_*p* > 1.0) and an average PSM ratio >2 (Log_2_ [PSM ratio OUT/control] > 1) between the purification from OUT and control cells. Among them, 104 proteins had a *p* value of medium stringency (*p* < 0.05, -Log_10_*p* > 1.3) and 27 proteins had a *p* value of high stringency (*p* < 0.01, -Log_10_*p* > 2) (Figures 4C, 4D, S5B, and S6; Table S2). There were substantial overlaps (148 and 82 proteins) between the Parkin substrate profiles generated by the OUT screen and the Lys-ε-diGly peptide enrichment from previous studies (Table S3).^19^^,^^71^ The OUT screen also identified many new Parkin substrates that have not been characterized before. Functional annotation-based bioinformatics analysis using DAVID showed that many substrates were involved in DNA replication, protein translation, rhythmic regulation, and intracellular protein transport (Figures 5A–5C). We also found numerous substrates that were assigned to central nervous system development and membrane vesicle-mediated transport clusters (Figures 5D and S7A). Moreover, disease-associated annotations related to Parkinsonism, neuropathy, neurodegeneration, intellectual disability, and glycogen storage disease were also identified (Figure S7B). Previous literature has demonstrated that disruptions in protein transport and vesicle trafficking mediated by Rab GTPases may play crucial roles in PD pathology.^14^^,^^72^^,^^73^^,^^74^ Therefore, we decided to verify Parkin-catalyzed ubiquitination of the Rab proteins from the OUT screen. Besides Rabs, cyclin-dependent kinase 5 (CDK5) was also found to be a potential substrate of Parkin from the OUT screen. Hyperactivity of CDK5 has been associated with neurotoxicity observed in PD, and it can suppress the E3 ligase activity of Parkin by phosphorylation.^75^^,^^76^ We thus also proceeded to verify CDK5 as a Parkin substrate in follow-up ubiquitination reactions.

### Validation of Parkin substrates by *in vitro* ubiquitination

**Figure 6:**
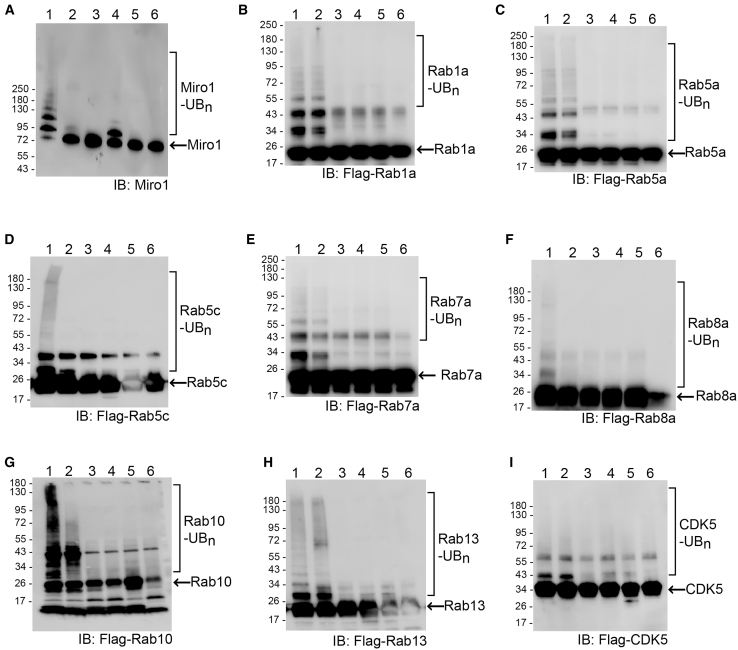
*In vitro* ubiquitination of Parkin substrates in reconstituted reactions (A–I) Reactions were set up with ATP and wt Uba1, UbcH7, and Parkin for the transfer of wt UB to Parkin substrates. PINK1 was also added to the reaction for the activation of Parkin by phosphorylating UB and Parkin. In control reactions, either PINK1 or each component of the UB transfer cascade of Parkin was excluded from the reaction to compare the activity of substrate ubiquitination in the presence of Parkin and PINK1. Lane assignments: 1, reaction with substrate proteins, PINK1 and the Uba1-UbcH7-Parkin cascade for the transfer of wt UB; 2, reaction missing PINK1; 3, reaction missing Uba1 as the E1; 4, reaction missing UbcH7 as the E2; 5, reaction missing Parkin as the E3; 6, reaction missing wt UB.

The OUT screen identified Rab7a as a high-fidelity substrate with a -Log_10_*P* of 2.01 and Rab1a, Rab5c, Rab10, Rab13, and CDK5 with -Log_10_*P* in the range of 1.1–1.3 (Figure S6 and Table S2). Rab8a was also identified in the OUT screen but with a nonsignificant *p* value. Rab5a was not identified by the OUT screen, but it is homologous to Rab5c from the OUT screen.^77^ We thus included Rab5a and Rab8a in the panel of Rabs for verification as Parkin substrates. We expressed and purified Rab GTPases from *E. coli* and set up *in vitro* ubiquitination reactions to verify their ubiquitination by wt Parkin (Figure 6). In the reconstituted reaction, we incubated the Rab proteins with wt UB and the cascade enzymes of Uba1-UbcH7-Parkin and probed the formation of UB-substrate conjugates. We also set up an *in vitro* ubiquitination reaction with the known Parkin substrate Miro1 as a positive control.^68^ We found Miro1, along with Rab1a, Rab5a, Rab7a, Rab10, and Rab13, could all be ubiquitinated by Parkin alone with the formation of substrate-UB conjugates, while the addition of PINK1 enhanced the ubiquitination of Miro1, Rab7a, Rab10, and Rab13 (Figures 6A–6C, 6E, 6G, and 6H). Rab5c mainly showed a mono-ubiquitination band with Parkin in the reaction alone, and the addition of Pink1 promoted its polyubiquitination (Figure 6D). Rab8a had a low activity with Parkin-catalyzed ubiquitination without PINK1 but formed polyubiquitinated species with PINK1 in the reaction (Figure 6F). In contrast, the addition of PINK1 showed little effect in enhancing the ubiquitination of Rab1a and Rab5a by Parkin in the reconstituted reactions, suggesting Parkin may catalyze the ubiquitination of these Rabs independent of PINK1 (Figure 6B and 6C). We also set up an *in vitro* ubiquitination reaction of CDK5 with Parkin and found Parkin could ubiquitinate CDK5 to form mono-ubiquitinated species alone or with the addition of PINK1 (Figure 6I). These results verified Rab proteins and CDK5 as Parkin substrates and PINK1 activation of Parkin enhanced the ubiquitination of some Rab proteins identified by the OUT screen.

### Verification of Parkin-catalyzed substrate ubiquitination in HEK293 cells and the regulation of substrate stability by Parkin

**Figure 7:**
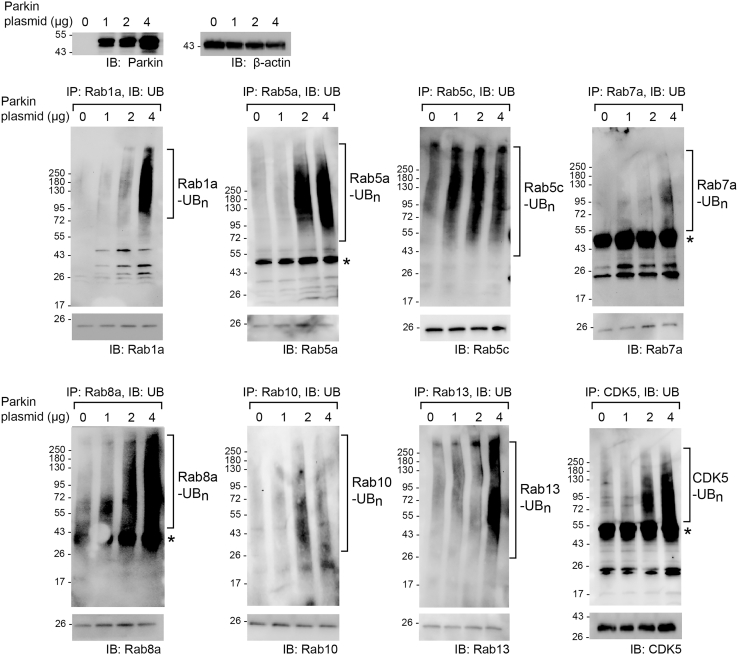
Validation of Parkin-induced ubiquitination of substrate proteins identified by the OUT cascade in cells Parkin was overexpressed with an N-terminal myc tag in HEK293 cells, and an increasing amount of Parkin plasmid was used for cell transfection. The N-terminal peptide fused to Parkin may perturb its auto-inhibited state and increase its activity without PINK1 activation in the cell.^69^ The designated substrates were immune purified from the cell with specific antibodies, and their ubiquitination levels were probed with an anti-UB antibody. Bands designated with a star on the western blots correspond to the size of IgG heavy chain.

**Figure 8:**
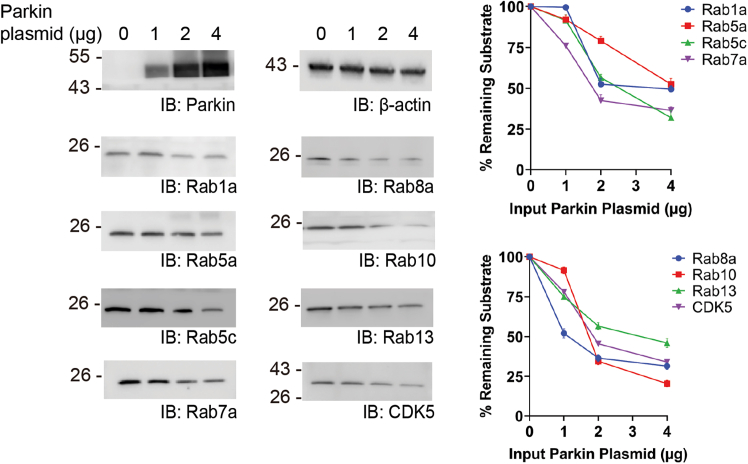
Suppressed cellular levels of Parkin substrates with increasing Parkin expression HEK293 cells were transfected with an increasing amount of Parkin plasmid, and the levels of designated substrates in the cell were assayed by western blots probed with substrate-specific antibodies. In the plots on the right, the relative intensities of the Parkin substrate bands were quantified and plotted with the increasing amount of Parkin plasmid used for transfection. The levels of the substrate proteins in the cell were mean ± S.E.M. of three independent experiments (*n* = 3).

We next verified the ubiquitination of identified Parkin substrates in HEK293 cells. We transfected cells with an increasing amount of Parkin plasmid and treated the cells with MG132 to inhibit proteasome activity. We then immune-purified substrate proteins and analyzed their ubiquitination. Increased Parkin expression in the cell enhanced substrate ubiquitination (Figure 7), demonstrating that Rab proteins and CDK5 are Parkin substrates. We also assayed the effect of Parkin-catalyzed ubiquitination on steady-state levels of substrates in the cell by increasing Parkin expression. As Parkin expression increased, Rab GTPase and CDK5 levels decreased (Figure 8), suggesting Parkin regulates the steady state level of these substrates by ubiquitin-mediated degradation in the cell. If Parkin regulates the stability of Rab proteins by ubiquitination, the suppression of Parkin activity in cancer cells may result in enhanced Rab expression. To verify this possibility, we examined the proteomic and genomic datasets available at the Clinical Proteomic Tumor Analysis Consortium (CPTAC) through the LinkedOmicsKB proteogenomics database (https://kb.linkedomics.org/). Our statistical analysis of the expression levels of Parkin, Rab1a, and Rab7a proteins in breast cancer tissues (*n* = 88) indicated that there were significant reciprocal correlations between Parkin and Rab1a, as well as between Parkin and Rab7a. By contrast, no significant correlations were identified in the mRNA levels of Parkin with those of Rab1a or Rab7a in the same breast cancer tissues (Figure S8). Therefore, Parkin expression is correlated inversely with Rab1a or Rab7a expression at the protein but not at the RNA level, which is consistent with posttranslational regulation of these Rab proteins by Parkin.

### Enhanced ubiquitination of Parkin substrates with stimulated mitophagy

**Figure 9:**
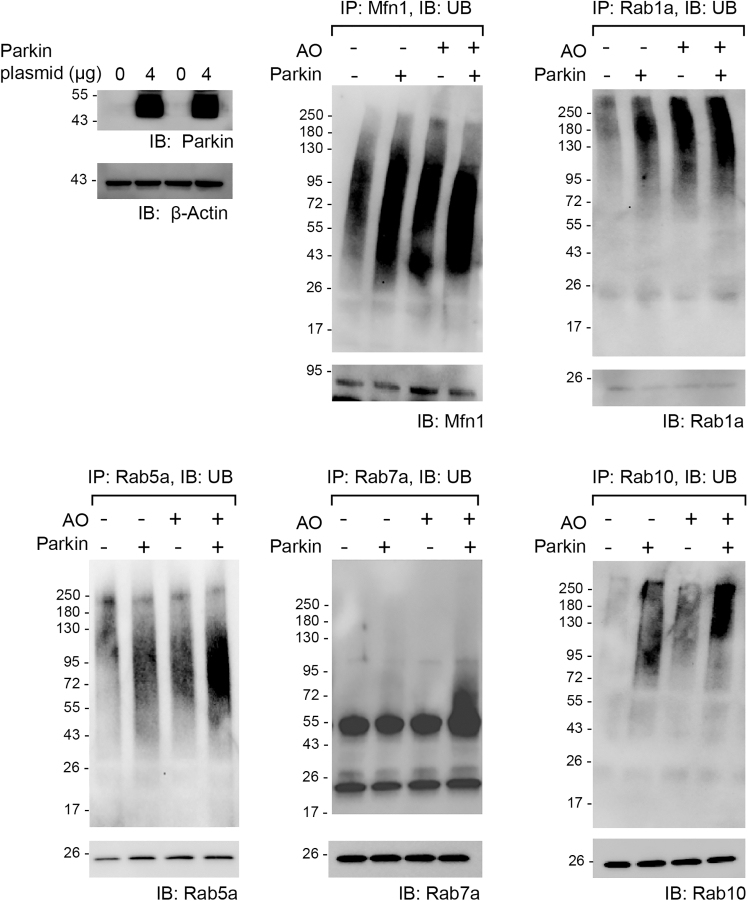
Induction of mitophagy led to enhanced ubiquitination of Parkin substrates HEK293T cells with and without transfection of the Parkin plasmid were treated with AO to induce mitophagy. Designated substrate proteins were immune purified with substrate-specific antibodies, and their ubiquitination levels were assayed by an anti-UB antibody.

The stimulation effect of PINK1 on Parkin-catalyzed Rab ubiquitination in reconstituted reactions (Figure 6) prompted us to assay if Parkin activation by PINK1 during mitophagy induction would lead to enhanced ubiquitination of substrates identified by the OUT cascade. We added antimycin and oligomycin (AO) to HEK293 cells to induce mitochondrial depolarization^7^ and assayed the ubiquitination of Parkin substrates along with Mfn1, which is known to be ubiquitinated by Parkin to initiate mitophagy.^21^ As validation of the assay, Mfn1 underwent enhanced ubiquitination with AO stimulation or with forced Parkin expression or under both conditions (Figure 9). Similarly, AO treatment significantly enhanced the ubiquitination of Rab1a, Rab5a, and Rab10 in cells without forced expression of Parkin. AO treatment combined with increasing amounts of Parkin expression further enhanced the ubiquitination of the assayed Rab proteins, including Rab1a, Rab5a, Rab7a, and Rab10. These results suggest that Parkin increases the ubiquitination of Rab proteins upon the induction of mitophagy in the cell.

## Discussion

In this study, we used phage display to engineer the RBR domain of Parkin so it can assemble an OUT cascade with the previously engineered xUba1-xUbcH7 pair for the exclusive delivery of HBT-xUB to Parkin and then to its substrates in the cell. Since the RBR domain binds UbcH7 and UB in the form of a thioester conjugate, we identified two interfaces in the Parkin RBR for restoring the interaction with xUB ∼ xUbcH7. One interface is between the loop in Ring1 and the N-terminal helix of UbcH7 that harbors the R5E and K9E mutation in xUbcH7, and another interface is between the β-stand of Ring2 and the R42 and R72 of UB that were both mutated to Glu in xUB. Phage selection of the RBR library with randomized loop residues in Ring1 failed to enrich clones with converged sequences to mediate xUB transfer. This suggests the interactions between the N-terminal helix of UbcH7 and the Ring1 domain of RBR would not play a decisive role in the association of the Parkin RBR with the UB ∼ UbcH7 conjugate. Instead, our modeled structure of Parkin RBR bound with UB ∼ UbcH5b based on the HOIP structure suggested key interactions between the β-stand of Ring2 and R42 and R72 of UB. Correspondingly, phage selection of the rat RBR library with randomized β-stand residues of Ring2 yielded consensus clones with restored activity for transferring xUB to Parkin RBR. Based on the phage selection results, we combined mutations E426R and N428R into the RBR domain of human Parkin to generate the engineered xParkin for the assembly of the OUT cascade. Thus, the combination of our modeling and phage selection results suggests R42 and R72 residues of UB constitute a key interface for the binding of UB ∼ UbcH7 conjugate with the catalytic active conformation of the RBR domain to mediate UB transfer to the RBR. Parkin has been shown to mediate E2-independent transfer of UB from E1 for self and substrate ubiquitination, suggesting the RBR domain of Parkin may directly bind to UB in the UB ∼ E1 conjugate to enable its transfer to the catalytic Cys residue of Parkin.^66^ Furthermore, the Ring2 domain of Parkin itself can mediate the transthiolation reaction between the UB ∼ UbcH7 conjugate and the catalytic Cys on Ring2, suggesting the interaction between Ring2 and the UB ∼ UbcH7 conjugate is sufficient for the UB transfer reaction.^78^ These studies match our observation that the interface between the Ring2 domain of Parkin RBR and the R42 and R72 residues of UB could be engineered for the transfer of xUB with the R42E and R72E mutations. Given the sequence of the β-strand region of the Ring2 domain of RBR E3s is highly conserved, it would be possible to transplant the E426R and N428R mutations from the Ring2 domain of xParkin to other RBR E3s for extending the OUT cascade to other RBR family members (Figure 1E).

The OUT screen identified 254 proteins as potential Parkin substrates (Table S2). Among them, 23 were found to be mitochondrial proteins based on their overlap with the human MitoCarta database (Table S3).^79^ Some of the mitochondrial proteins identified in the OUT screen are known Parkin substrates, including APEX1, HSD17B10 and VDAC2.^19^^,^^80^^,^^81^^,^^82^ So far, several studies have been reported on the identification of Parkin substrates in various cell types, including cancer and neuronal cells. The majority of these studies used an anti-Lys-ε-diGly antibody to enrich trypsinized peptides containing a diGly-Lys conjugate as a remnant of UB modification to reveal the difference in the protein ubiquitome with or without Parkin expression or mitophagy induction. Proteins with enhanced ubiquitination due to Parkin expression or activation during mitophagy were assigned as potential substrates. In a pioneering study by Sarraf et al., proteins with enhanced ubiquitination were identified in HCT116, HeLa, and SH-SY5Y cells with Parkin expression and chemical stimulation of mitophagy to assemble a substrate profile of Parkin, in which 148 common substrates were found matching the Parkin substrate profile identified by OUT (Table S3).^19^ In another study by Agarwal et al., a Parkin substrate profile in prostate cancer PC3 cells was identified under the condition of enhanced Parkin expression without mitophagy induction, and it showed 82 common substrates with the Parkin substrate profile from the OUT screen (Table S3).^71^ The substantial overlap between the Parkin substrate profiles from the OUT screen and Lys-ε-diGly proteomics validates the use of the Parkin OUT cascade for substrate identification. We also analyzed data from other studies that enriched Lys-ε-diGly peptides from neuronal cells with Parkin expression and mitophagy induction. We found 4 targets in the Ordureau et al. study using induced neurons from human embryonic stem cells and 12 targets in the Antico et al. study using mouse cortical neurons overlapping with the OUT-substrate profile of Parkin (Table S3).^58^^,^^59^ The smaller number of overlapping Parkin substrates identified in neuronal cells and HEK293 cells may suggest a greater difference in proteome composition between the two cell types. Another study identified Parkin substrates by Parkin-mediated transfer of wt UB in Drosophila, and four overlapping substrates were identified when compared to the Parkin substrate profile from the OUT screen (Table S3).^56^

Based on the OUT screen, we found Parkin can ubiquitinate a panel of Rab proteins, including Rab1a, Rab5a, Rab5c, Rab7a, Rab8a, Rab10 and Rab13, and regulate their stability in the cell. Rabs are GTPases that are prenylated at the C-terminus to anchor them on membrane structures, including plasma membrane, endosomes, ER, and Golgi.^77^ Rab proteins define the identity of the cell membrane and program their functions in subcellular trafficking, sorting, recycling and degradation. Among the Rabs identified as Parkin substrates, Rab5a and Rab7a are endocytic Rabs that reside on early and late endosomes, respectively, to register them for degradation by fusion with the lysosome or for recycling by directing them to the trans-Golgi network (TGN) or the plasma membrane. Rab1a, Rab8a and Rab10 are exocytic Rabs that mediate anterograde transport of vesicles from the ER to the plasma membrane.^83^^,^^84^ Rab1a, Rab5a, Rab7a, and Rab10 have also been identified to play important roles in mitophagy. Upon PINK1 deposition to depolarized mitochondria to activate UB chain synthesis by Parkin, RABGEF1, a Rab5 GEF, binds to conjugated UB chains on OMM proteins for recruiting Rab5 to damaged mitochondria. Then, a Mon1-Ccz1 complex facilitates the replacement of Rab5 with Rab7 on the mitochondria surface, and Rab7 engages the Atg9-bearing vesicles from the TGN or late endosome as the membrane source for autophagosome assembly.^85^^,^^86^^,^^87^ Rab1 is anchored on the Atg9 vesicles for binding to the ULK complex and PI3KC3/VPS34 complex to prime the formation of the omegasome, the precursor of the autophagosome.^88^^,^^89^^,^^90^ Furthermore, the recruitment of Rab10 to damaged mitochondria is dependent on PINK1 and Parkin activities. Rab10 promotes the association of the adaptor protein Optineurin (OPTN) with damaged mitochondria for mitophagy initiation.^91^ Beyond autophagosome formation, Rab7a could bind to effector proteins such as RPIL to transport autophagosomes to perinuclear regions for their fusion with the lysosome.^92^ Also, Parkin-catalyzed ubiquitination on damaged mitochondria can tether the mitochondria with Rab5-anchored early endosomes that eventually fuse with the lysosome to undergo mitophagy-independent degradation.^93^ In this study, we found Parkin ubiquitinated a set of Rabs (Rab1a, Rab5a, Rab7a, and Rab10) that play crucial roles in mitophagy. We also found that the induction of mitophagy in the cell enhanced Parkin-catalyzed ubiquitination of Rab proteins. These results suggest Parkin may affect Rab-mediated autophagosome assembly and lysosome processing besides its well-characterized role in UB chain synthesis on damaged mitochondria. Further work is warranted to reveal the effects of Parkin-catalyzed Rab ubiquitination on mitophagy.

Genetic analysis of PD patients substantiates the association of PD with deficits in endosome trafficking and mitophagy pathways in the cell and both pathways hinge on the correct functioning of a suite of Rabs.^72^^,^^73^ For example, Rab5a and Rab7a are anchored on endosomes and, like autophagosome assembly, endosomal maturation is accompanied by a Rab5a to Rab7a conversion.^94^^,^^95^^,^^96^ The subsequent trafficking and fusion of late endosomes with the lysosome are mediated by the Rab7a-RPIL complex. Both Rab5a and Rab7a were found to be regulated by ubiquitination during membrane trafficking. In fact, Rab7a has been identified as a Parkin substrate,^97^^,^^98^ and Parkin-catalyzed monoubiquitination of Rab7a at K38 can enhance the tethering between mitochondria and the lysosome in neuronal cells for the flux of amino acids to mitochondria for metabolic processing.^99^ Monoubiquitination of Rab7a at K191 by an unidentified E3 was found to weaken the binding of RPIL with Rab7a and stall the fusion of the late endosome to the lysosome.^100^^,^^101^ Similarly, monoubiquitination of Rab5 by an unknown E3 can affect the association of Rab5 with the endosome, decreasing receptor endocytosis.^102^ Our results confirm Parkin can ubiquitinate both Rab5a and Rab7a, suggesting Parkin may broadly alter endosomal trafficking in cells. Beyond mitophagy, it would be of interest to further characterize the role of Parkin in endosome regulation to reveal potential linkages between PD-associated Parkin mutations and endosomal malfunction.

The identification of Rabs as Parkin substrates may suggest Rabs as a nexus linking Parkin with other PD-causing genes such as *LRRK2* and *SNCA* encoding leucine-rich repeat kinase 2 (LRRK2) and α-synuclein, respectively. Rab5a, Rab8a and Rab10 are among the Rabs that are phosphorylated by LRRK2, the mutations of which lead to enhanced activity in individuals with familial PD.^103^^,^^104^^,^^105^ Phosphorylation of membrane-anchored Rab8a by LRRK2 enhanced the membrane deposition of LRRK2 that would increase its phosphorylation of Rab10,^106^ and the phosphorylation of both Rabs by LRRK2 on the lysosome surface promotes secretion of lysosome contents for stress release.^107^^,^^108^ On the other hand, Rab8a and Rab10 with enhanced phosphorylation catalyzed by PD-related LRRK2 mutants showed increased binding with effector proteins (RILPL1 and RILPl2), which inhibit protein trafficking and hinder ciliogenesis and neuronal cell development.^109^^,^^110^ Also, excessive phosphorylation of Rab8a and Rab10 due to LRRK2 mutation inhibits the binding of Rabs with the GDP dissociation inhibitor (GDI) that is responsible for extracting the GDP-bound Rab from the membrane. This would impair the proper recycling of Rab proteins in autophagy and lysosome pathways and may account for the accumulation of α-synuclein in PD.^105^^,^^111^ The PD-related LRRK2 mutation may also affect mitophagy through Rab10. It has been shown that LRRK2-mediated phosphorylation of Rab10 inhibits its interaction with the mitophagy adaptor OPTN, preventing OPTN recruitment to damaged mitochondria.^91^ In addition, LRRK1, a paralog of LRRK2, and TBK1, a kinase phosphorylating OPTN to enhance its binding with UB chains on mitochondria, have been found to phosphorylate Rab7a to facilitate the recruitment of Atg9-bearing vesicles for autophagosome assembly.^112^^,^^113^^,^^114^ In our study, we found Parkin can ubiquitinate Rab5a, Rab7a, and Rab10 in response to mitophagy induction in the cell. Further studies on the cross-regulation of ubiquitination by Parkin and phosphorylation by LRRK1/2 and TBK1 on Rab proteins may reveal another layer of control for mitophagy initiation.

The membrane transport processes mediated by the Rab proteins also operate in neuronal cells to enable specialized functions, including neurite outgrowth, receptor signaling, and the transport and recycling of synaptic vesicles.^115^^,^^116^ Parkin is known to regulate the levels of the dopamine transporter and AMPA and NMDA receptors at the neuronal synapse as well as the trafficking and endocytosis of presynaptic vesicles.^29^^,^^117^^,^^118^^,^^119^^,^^120^^,^^121^^,^^122^^,^^123^ So, Parkin-catalyzed Rab ubiquitination may underpin its regulation of synaptic activities. The Rab proteins verified as Parkin substrates in this study also have oncogenic potential, and their elevated expression has been found in various types of cancer cells. For example, Rab5 and Rab7 affect the endocytosis and intracellular transport of EGFR, and high levels of the two Rabs enable the cell to acquire invasive features such as focal adhesion disassembly and epithelial-mesenchymal transition.^124^^,^^125^^,^^126^^,^^127^^,^^128^ Rab13 controls protein trafficking pathways pertaining to cancer growth, including integrin recycling for cell migration and the membrane transport of GLUT4 and VGEFR for glucose uptake and angiogenesis.^129^ Our findings that Parkin mediates ubiquitination of these oncogenic Rabs and decreases their expression in the cell, as well as the reciprocal correlations between Parkin and Rab1a or Rab7a in breast cancer tissues, reiterate the role of Parkin as a tumor suppressor. We also verified CDK5 as a Parkin substrate. The hyperactivation of CDK5 is linked to PD for its role in promoting oxidative stress and mitochondrial dysfunction in dopaminergic neurons, leading to neuronal inflammation and apoptosis.^130^^,^^131^^,^^132^ The identification of Parkin-mediated CDK ubiquitination and reduction in the cellular level match the neuroprotective role of Parkin, which could be further verified.

Overall, our work developed an OUT cascade for profiling Parkin substrates in the cell. The verification of Parkin-catalyzed ubiquitination of the Rab GTPase panel and CDK5 from the OUT screen suggests the potential roles of Parkin in regulating membrane vesicle trafficking and associated pathways implicated in neurodegeneration. Our study validated the OUT cascade of Parkin as an empowering tool to map E3 substrates and established a bridgehead for extending the OUT cascade to other RBR E3s to decipher their cellular functions.

### Limitations of the study

A limitation of our study is that we performed the OUT screen of Parkin substrates in HEK293 cells, which are drastically different from neuronal or cancer cells in cellular function and proteome composition. Future work on applying the OUT cascade of Parkin to disease-relevant cell types may reveal pathogenic pathways directly related to Parkin-catalyzed substrate ubiquitination. Another limitation is that we overexpressed xParkin and HBT-xUB in the cells to label potential Parkin substrates by the OUT cascade. Overexpression of xParkin might result in the transfer of HBT-xUB to non-specific targets. Incorporating the xParkin mutations into the endogenous *PRKN* gene by genome editing may solve this issue.

## Resource availability

### Lead contact

Further information and requests for most resources and reagents should be directed to and will be fulfilled by the lead contact, Jun Yin (junyin@gsu.edu).

### Materials availability

Cells and plasmids generated in this study are available from the lead contact upon completion of a material transfer agreement.

### Data and code availability

• All other data reported in this work are available by the lead contact upon request.
• The proteomics data for the analysis of Parkin substrates from the OUT screen are listed in Tables S1 and S2 in the supplemental information. The original mass spectrometry data for the identification of Parkin substrates have been deposited to the ProteomeXchange Consortium via the PRIDE partner repository^133^ with the dataset identifier PXD063080.
• This study does not report any original code.

## Acknowledgments

This work was supported by the National Institute of Health (R01GM104498 to J.Y. and H.K., R21NS116760 to J.Y. and A.M.M., and R35GM139382 to I.I.), National Science Foundation CAREER Award (2047700) to A.M.M., and National Science Foundation grants (2109051 to J.Y. and MCB-2027902 to I.I.). This work was also supported by the Natural Science Foundation of China (31971187 to B.Z.) and the Science and Technology Commission of Shanghai Municipality Project (20JC1411200 to B.Z.). L.Z. was supported by a graduate fellowship from the Center for Diagnostics and Therapeutics of Georgia State University. We thank Chunli Yan for helping to prepare the STAR Methods section of the manuscript related to molecular modeling.

## Author contributions

J.Y., B.Z., and H.K. conceived the project. S.F. performed the proteomics screen of the Parkin substrates. L.Z., J.Z., X.W., I.H.J., S.E.J., G.H.J., and Y.X. verified Parkin substrates by ubiquitination assays *in vitro* and in the cell. G.C. and S.L. engineered Parkin RBR to generate the OUT cascade by phage display. B.R.K. and I.I. generated the structural model of Parkin RBR in complex with UB ∼ UbcH5b. D.D. and N.T.S. guided proteomic identification of Parkin substrates from the OUT screen. W.W. and A.M.M. performed bioinformatic analysis of Parkin substrates. J.Y., B.Z., H.K., A.M.M., S.F., L.Z., G.C., and W.W. prepared the manuscript with input from all the authors. All authors analyzed and interpreted the results and commented on the manuscript.

## Declaration of interests

The authors declare that they have no competing interests.

## STAR★Methods

### Key resources table

**Table:** 

REAGENT or RESOURCE	SOURCE	IDENTIFIER
**Antibodies**		

anti-CDK5 antibody	Santa Cruz Biotechnology	Cat# sc-6247
anti-myc antibody	Santa Cruz Biotechnology	Cat# sc-40
anti-Rab5a antibody	Santa Cruz Biotechnology	Cat# sc-166600
anti-Rab7 antibody	Santa Cruz Biotechnology	Cat# sc-376362
anti-V5 antibody	Santa Cruz Biotechnology	Cat# sc-271944
anti-UB antibody	Santa Cruz Biotechnology	Cat# sc-8017
anti-beta Actin antibody	Santa Cruz Biotechnology	Cat# sc-47778
anti-Rab1a antibody	ABclonal	Cat# A14663
anti-Rab5c antibody	ABclonal	Cat# A20882
anti-Rab8a antibody	ABclonal	Cat# A20976
anti-Rab10 antibody	ABclonal	Cat# A4459
anti-Rab13 antibody	ABclonal	Cat# A10571
anti-Flag M2 antibody	Sigma-Aldrich	Cat# F3165
F(ab’)2-Goat anti-Mouse IgG (H + L) Cross-Adsorbed Secondary Antibody, HRP	Thermo Fisher Scientific	Cat# 31438
anti-rabbit IgG-HRP (goat)	Santa Cruz Biotechnology	Cat# sc-2004

**Bacterial and virus strains**		

Escherichia coli strain XL1-Blue	Agilent Technologies	Cat# 200228
*E. coli* strain SS320	Amid Biosciences	Cat# SS320-201
E. coli strain BL21(DE3)	Invitrogen	Cat# EC0114
M13KO7 helper phage	Invitrogen	Cat# 18311-019

**Chemicals, peptides, and recombinant proteins**		

High-glucose Dulbecco’s modified Eagles medium (DMEM)	Life Technologies	Cat# 11965092
Fetal bovine serum (FBS)	Life Technologies	Cat# 11965092
Doxycycline	Thermo Scientific Chemicals	Cat# 24390-14-5
Hygromycin	Gibco	Cat# 10687010
Blasticidin	Thermo Fisher Scientific	Cat# A1113902
Zeocin	Thermo Fisher Scientific	Cat# R25005
Puromycin	Thermo Fisher Scientific	Cat# A1113802
Antimycin	Sigma-Aldrich	Cat# A8674
Oligomycin	Sigma-Aldrich	Cat# 75351
Taq DNA polymerase	Promega	Cat# M3001
Ni SepharoseÔ High Performance	GE Healthcare	Cat# 17-5268-01
Pierce^TM^ high-capacity streptavidin agarose resin	Thermo Scientific	Cat# 20359
Protein A/G PLUS-agarose	Thermo Fisher Scientific	Cat# 20241
Parkin protein	R&D Systems	Cat# E3-160-025
PINK1 protein	R&D Systems	Cat# NBP1-72481

**Critical commercial assays**		

ViraPower Lentiviral Packaging Mix	Life Technologies	Cat# K4975-00
DNA Maxiprep kit	Qiagen	Cat# 12162
DharmaFECT kb DNA transfection reagent	Horizon Discovery	Cat# T-2006-01
Trans-Blot® Turbo™ RTA Mini PVDF Transfer Kit	Bio-Rad	Cat# 1704272
SuperSignal™ West Pico PLUS Chemiluminescent Substrate	Thermo Fisher Scientific	Cat# 34580

**Deposited data**		

MS proteomics data of HBT-xUB-conjugated proteins from affinity purification of OUT cells and control cells	This study	ProteomeXchange with identifier PXD063080

**Experimental models: Cell lines**		

HEK293T cells	American Tissue Culture Collection (ATCC)	Cat# CRL-3216

**Oligonucleotides**		

Primer GC1: GACAAGCCGCGGA AAGACACCTCTGTTGCG	Integrated DNA Technologies	N/A
Primer GC2: CTGGCCACTAGTA ACGTCGAACCAGTGGTC	Integrated DNA Technologies	N/A
Primer GC3: CGGAACGTTGCAACG	Integrated DNA Technologies	N/A
Primer GC4: CGTTGCAACGTTCC GGCTGCTAAAGCTGCTGGTTGC ATGCACATG	Integrated DNA Technologies	N/A
Primer GC5: CGTTGCAACGTT CCGNNKNNKAAANNKNNKGG TTGCATGCACATG	Integrated DNA Technologies	N/A
Primer GC6: TCTGTTGGATCCAAAG ACACCTCTGTTGCG	Integrated DNA Technologies	N/A
Primer GC7: ACGATGCGGCCGCCT AAACGTCGAACCAGTG	Integrated DNA Technologies	N/A
Primer GC8: CACCCGACCTCTG ACAAAGACACCTCTGTT	Integrated DNA Technologies	N/A
Primer GC9: TCTGTTGGATC CATGATCGTTTTCGTTCG	Integrated DNA Technologies	N/A
Primer GC10: AACAGAGGTGT CTTTGTCAGAGGTCGGGTG	Integrated DNA Technologies	N/A
Primer SF19: TACTTATTCGAAG CGGCCGCCTACACGTCGAACCAG	Integrated DNA Technologies	N/A
Primer SF20: AAGCGAGGATCC GCTAGCATGATAGTGTTTGTC	Integrated DNA Technologies	N/A
Primer SF22: CACTGGTACAT GGCAGCGGGGAC	Integrated DNA Technologies	N/A
Primer SF23: TGCCATGTAC CAGTGAGGAAAAGGGG AGGCTGCATGCAC	Integrated DNA Technologies	N/A
Primer SF30: AAGCGAGGAT CCGCTAGCAAGGAAACACCAGTAGC	Integrated DNA Technologies	N/A
Primer SF31: TGCCATGTACCAG TGAGGAAAAGGGGAGGCGCCATGCAC	Integrated DNA Technologies	N/A
Primer SJ1: GGACGGCCGCG GATGCAGAAATACGAGAAACTGG	Integrated DNA Technologies	N/A
Primer SJ2: GGACCGCCGCGGA TGGCTAGTCGAGGCGCAACAAGACC	Integrated DNA Technologies	N/A
Primer SJ3: GGACGGCCGCGG ATGTCCAGCATGAATCC	Integrated DNA Technologies	N/A
Primer SJ4: GGCAGGGCGGCC GCTTAGCAGCAACCTCC	Integrated DNA Technologies	N/A
Primer SJ5: GGACCGCCGCGG ATGGCTAGTCGAGGCGCAACAAGACC	Integrated DNA Technologies	N/A
Primer SJ6: GGCAGGGCGGCC GCTTAGTTACTACAACA	Integrated DNA Technologies	N/A
Primer SJ7: GATCGTCCGCGG ATGGCGGGTCGGGGAGGCGC	Integrated DNA Technologies	N/A
Primer SJ8: GACATTGCGGCCG CCTAGTTGCTGCAGCACTGGCTCC	Integrated DNA Technologies	N/A
Primer SJ9: GGACGGCCGCGG ATGACCTCTAGGAAGAAAGTGTTGC	Integrated DNA Technologies	N/A
Primer SJ10: GGCAGGGCGGCC GCTTAGCAACTGCAGCT	Integrated DNA Technologies	N/A
Primer SJ11: GAATTCCCGCGGA TGGCGAAGACCTACGATTAC	Integrated DNA Technologies	N/A
Primer SJ12: CGCGCTGCGGCC GCCTAATCTCTGGCGAGAGTG	Integrated DNA Technologies	N/A
Primer SJ13: GGACCGCCGCGG ATGGCGAAGAAGACGTACG	Integrated DNA Technologies	N/A
Primer SJ14: GGCAGGGCGGC CGCTCAGCAGCATTTGCT	Integrated DNA Technologies	N/A
Primer SJ15: GACGCTCCGCG GATGGCCAAAGCCTACGACCACC	Integrated DNA Technologies	N/A
Primer SJ16: CAGACTGCGGCC GCTCAGCCCAGGGAGCACTTGTTGG	Integrated DNA Technologies	N/A
Primer Jun13: ACTTTATGCTTC CGGCTCGTATGT	Integrated DNA Technologies	N/A
Primer Jun14: AATCAAAATCA CCGGAACCAGAGCC	Integrated DNA Technologies	N/A

**Recombinant DNA**		

pLenti-puro-ARID1A	Addgene	39478
pEGFP-C1-Rab1a	Addgene	49467
pcDNA5/FRT/TO-Flag-Rab5a	Addgene	28043
pBS-L30-mRuby3-Rab5c	Addgene	166426
pcDNA3-EGFP-Rab7a	Addgene	28047
pEGFP-C2-Rab8a	Addgene	86075
pEGFP-C1-Rab10	Addgene	49472
pEGFP-C1-Rab13a	Addgene	49548
pGEX-2TK-CDK5	Addgene	24894
pRK-myc-human Parkin	Addgene	17612
pET-wtUB, pET-wtUba1, pET-wtUbcH7	Wang et al. 2017^47^	N/A
pET-xUB, pET-xUba1, pET-xUbcH7	Wang et al. 2017^47^	N/A
pLenti-xUB, pLenti-xUba1, pLenti-xUbcH7	Wang et al. 2017^47^	N/A
pLenti-xParkin, pLenti-xParkinC431A	This study	N/A
pComb3H-Parkin RBR domain	Bhuripanyo et al. 2018^48^	N/A

**Software and algorithms**		

ImageJ	NIH	http://imagej.nih.gov
R	The R Project for Statistical Computing	https://www.r-project.org/
Python	Python	https://www.python.org/

### Experimental model and study participant details

#### Cell lines and helper phage

The mammalian and bacterial cells and helper phage used in the study are listed in the key resources table. HEK293T cells (Cat# CRL-3216) were acquired from and authenticated by the American Tissue Culture Collection (ATCC). The cells were cultured at 37°C in DMEM medium supplemented with 10% Fetal Bovine Serum (FBS, Gibco) in a humidified atmosphere containing 5% CO_2_. The influence of sex/gender of the cell was not applicable to this study. *Escherichia coli* strains used in this study included XL1-Blue for cloning, SS320 for phage production, and BL21(DE3) for protein expression. Stocks of the *E.* *coli* cells were stored at −80°C in 15% glycerol, and the cells were cultured at 30 or 37°C in Luria-Bertani (LB) or 2× Yeast Extract Tryptone (2×YT) medium with proper antibiotics such as kanamycin or ampicillin as detailed in the STAR Methods section. M13KO7 helper phage (Cat# 18311-019) was from Invitrogen and used to infect SS320 cells to generate phage for displaying the Parkin RBR domain library. The stocks of the helper phage solution were stored at −80°C before use.

### Method details

#### Construction of the protein expression plasmids

The genes of full-length human Parkin were amplified by polymerase chain reactions (PCR) with primers SF19 and SF20. The amplified fragments were digested by restriction enzymes BamHI and NotI and cloned into the pGEX-4T1 vector. The genes of the Parkin RBR domain (residues 219–465) were amplified by PCR with primers SF19 and SF30. The amplified fragments were also digested with BamHI and NotI, and cloned into the pGEX-4T1 plasmid. For the expression of full-length Parkin with mutated RBR domains, mutant RBR genes were amplified with primers SF19 and SF20 and cloned into the pGEX-Parkin vector between restriction sites BamHI and NotI. To characterize the mutant Parkin RBR domains from phage selection, the genes of the mutant RBR domains were amplified by PCR from the pComb vectors with primers SF19 and SF30. The amplified DNA fragments were digested by BamHI and NotI, and cloned into pGEX vector. The gene of full-length human Parkin was cloned into pLenti-vector between the BstbI and NheI restriction sites with primers SF19 and SF20. The Parkin mutant with E426R and N428R mutations were introduced into wt Parkin by primers SF19, SF22, SF23, SF20 with overlapping PCR. The assembled PCR fragments were digested by BstbI and NheI and cloned into the pLenti vector. To construct the catalytically inactive Parkin mutant, overlapping PCR was performed with primers SF19, SF22, SF31, and SF20 for introducing the C431A mutation into pLenti-xParkin. For the expression of Parkin substrates, the CDK5 gene was amplified from pGEX-CDK5 plasmid with primers SJ1 and SJ2, Rab1a amplified with SJ3 and SJ4, Rab5a with SJ5 and SJ6, Rab5c with SJ7 and SJ8, Rab7a with SJ9 and SJ10, Rab8a with SJ11 and SJ12, Rab10 with SJ13 and SJ14, and Rab13 with SJ15 and SJ16. The PCR fragments were digested with SacII/NotI and cloned into the pET-28a plasmid. The pET and pGEX plasmids were transformed into BL21(DE3) pLysS chemical competent cells (Invitrogen) for protein expression.

#### Construction of a structure model of the human Parkin RBR-UbcH5b-UB complex

For modeling the human Parkin-UbcH5b-UB complex, we employed the structure of the human HOIP-RBR/UbcH5b-UB complex (PDB ID: 5EDV).^64^ Initially, homology models of the human Parkin dimer (residues 228–378 and 393–464) were generated using the structure of HOIP RBR (PDB ID: 5EDV)^64^ as a template. The model was then superimposed onto the 5EDV structure to obtain the human Parkin-UbcH5b-UB complex. Finally, structural minimization, including the adjustment of side chains, was performed to optimize their orientations using the Rosetta Relax protocol implemented in ROSETTA.^134^

#### Displaying the Parkin RBR domain on M13 phage

The RBR domain of the rat Parkin (residues 219–465) was amplified by PCR with primers (GC1 and GC2) and cloned into the phagemid vector pComb3H between the SacII and SpeI restriction sites to display the RBR on phage with an N-terminal FLAG tag and as a fusion to the phage pIII protein at the C-terminus. The preparation of the RBR displayed phage was performed following the protocol reported previously.^53^ Briefly, *E. coli* SS320 cells were transformed with the pComb3H phagemid vector, cultured in the 2×YT media, and infected with M13KO7 helper phage. The infected cell culture was shaken at 37°C overnight in 100 mL 2×YT that was supplemented with 100 μg/mL ampicillin and 50 μg/mL kanamycin. The next day, the cells were pelleted by centrifugation, and the phage particles in the supernatant were PEG precipitated and resuspended in TBS buffer (20 mM TrisHCl, 150 mM NaCl, pH 7.5). Phage titrations were performed with *E. coli* XL1-blue cells with standard procedures.^135^^,^^136^ Phage displaying Parkin library for selection and SV5V for model selection were prepared following the same procedure. The display of the RBR domain on phage was confirmed by western blotting probed with an anti-Flag antibody. To confirm the catalytic activity of the RBR domain displayed on phage surface, ubiquitination reactions were set up with 2 × 10^10^ phage particles, 0.5 μM Uba1, 5 μM UbcH5b, and 20 μM HA-wt UB in a reaction buffer with 50 mM Tris-HCl (pH 7.5), 10 mM MgCl_2_, 5 mM ATP, and 50 μM dithiothreitol (DTT). After 1-h incubation at 37°C, the reaction mixture was analyzed by SDS–polyacrylamide gel electrophoresis (PAGE) and western blotting probed with an anti-HA antibody to detect the conjugation of HA-UB to the RBR domain on phage or with an anti-Flag antibody to detect the ubiquitinated RBR-pIII fusion.

#### Model selection of phage-displayed Parkin RBR

UB transfer reaction with the phages was set up in a total volume of 100 μL with 0.5 μM wt Uba1, 5 μM wt UbcH7, and 5 μM biotin–wt UB in a reaction buffer containing 50 mM Tris-HCl (pH 7.5), 10 mM MgCl_2_, 5 mM ATP, and 50 μM DTT,. 1 × 10^11^ phage mixtures with Parkin and SV5V-displayed phages mixed at a ratio of 1/10, 1/100, or 1/1000 in TBS buffer (pH 7.5) were then added to the reaction. The reaction was allowed to proceed for 1 h at room temperature before it was added to 400 μL 3% BSA in TBS buffer (pH 7.5). 100 μL of phage solution was distributed to each well of a 96-well plate coated with streptavidin. The streptavidin plate was incubated at room temperature for 1 h. The supernatant was discarded, and the plate was washed 30 times with 0.05% (v/v) Tween 20, 0.05% (v/v) Triton X-100 in TBS and 30 times with TBS, each time with 200 μL of solution per well. After washing, phage particles bound to the streptavidin surface were eluted by adding 100 μL of 10 mM DTT in TBS to each well. Eluted phages were combined, added to 10 mL of log phase *E. coli* XL1-Blue cells, and shaken at 37°C for 1 h to infect the cells. The cells were then plated on LB agar plates supplemented with 2% (w/v) glucose and 100 μg/mL ampicillin. After overnight incubation at 37°C, colonies on the plates were analyzed by colony PCR with primers Jun13 and Jun14. To set up colony PCR, 40 μL reaction mixture for each PCR reaction was prepared, containing 29.4 μL H_2_O, 4 μL Mg^2+^ free PCR buffer (Promega), 4 μL 25 mM MgCl_2_, 1 μL Jun13 primer (10 μM), 1 μL Jun14 primer (10 μM), 0.32 μL dNTP mix (25 mM each dNTP) and 0.2 μL Taq DNA polymerase (Promega). Pipette tips were used to transfer colonies from the agar plates to the PCR reaction mixture. The PCR reactions were run with the following program: 95°C for 10 min, then 35 cycles of 94°C for 1 min, 50°C for 45 s, 72°C for 1.5 min, and a final step of 72°C for 5 min. For analysis, 10 μL of the PCR reaction was loaded on a 1% agarose gel, and the PCR products were separated by electrophoresis.

#### Construction of the RBR library of Parkin for phage display

Residues to be randomized in the Parkin RBR (I425, E426, N428, and G429) were first mutated to Ala to generate phagemid pComb-Parkin-RBR-4Ala. For mutagenesis, the RBR gene was amplified by two sets of primers, GC1/GC3 and GC4/GC2. The PCR fragments were assembled by overlap extension for cloning into the pComb phagemid between restriction sites SacII and SpeI. For randomization of the designated residues in the Parkin RBR, pComb-Parkin-RBR-4Ala was used as the template for PCR reactions with primer pairs GC1/GC3 and GC5/GC2. GC5 primer contained NNK codons for replacing I425, E426, N428, and G429 with randomized residues in the Parkin RBR. The overlap extension of the amplified fragments was cloned into pComb phagemid between restriction sites SacII and SpeI to generate the phagemid library. The library DNA was transformed into XL1-Blue cells by electroporation. The cells were plated on LB-agar plates containing ampicillin (100 μg/mL) and glucose (2%) and incubated overnight at 37°C. A total of 10 transformation reactions were performed each time with 50 μL of competent cells. The transformation efficiency of the library DNA was titered, and the size of the library was estimated to be 2× 10^7^, exceeding the theoretical diversity of the Parkin library with four randomized sites (1.6 × 10^5^). After incubation, the cells growing on the plate were scrapped, and library phagemid DNA was prepared with a DNA Maxiprep kit (Qiagen).

#### Phage selection of the Parkin RBR library

For preparing phages displaying the Parkin library, 1 μL of the library phagemid DNA was used to transform 100 μL electrocompetent XL1Blue cells. After electroporation, the cell culture was shaken at 37°C overnight in 100 mL 2×YT that was supplemented with 100 μg/mL ampicillin and 50 μg/mL kanamycin. The next day, the cells were pelleted by centrifugation, and the phage particles in the supernatant were PEG precipitated and resuspended in TBS buffer (20 mM TrisHCl, 150 mM NaCl, pH 7.5). Phage titrations were performed with E. coli XL1-blue cells with standard procedures.^135^^,^^136^ For the first round of phage selection, 10^10^ phage particles were reacted with 0.5 μM xUba1, 10 μM xUbcH7, and 0.5 μM biotin-xUB for 1 h. Reactions were diluted 10-fold into 0.1% bovine serum albumin (BSA)–TBST (20 mM TrisHCl, 150 mM NaCl, 0.1% Tween 20, pH 7.5) and bound to a streptavidin plate for 1 h at room temperature. The plate was washed 30 times with 0.1% BSA-TBST, and then 100 μL TBS containing 10 mM DTT was added to each well to elute the phage particles. Each 100 μL of the phage elution solution was added to a culture of 1 mL XL1-Blue cells growing at the log phase, and the culture was shaken slowly for 2 h at 37°C to enable phage infection of the cell. Then, the cells were plated onto LB-agar plates containing carbenicillin (100 μg/mL) and glucose (2%). After overnight incubation at 37°C, the colonies on the plates were collected, and the phagemid DNA was extracted with a DNA Miniprep kit. In parallel, control reactions were set up with xUba1, xUbcH7 or biotin-xUB missing from the reaction mixture. Phages in the control reactions were also bound to the streptavidin plate and eluted with TBS-DTT solution. The number of phages from the selection and control reactions were titered to follow the enrichment of the phage particles from the selection reaction. After each round of selection, 10–20 colonies were picked from the LB-agar plate of the selection reaction to prepare phagemid DNA. DNA from each isolated colony was sequenced to determine if the RBR library was converged to a consensus sequence. The library phagemid from the previous round of selection was used for the next round of phage preparation. The concentration of xUba1 and xUbcH7 enzymes and biotin-xUB was decreased in each round of selection reaction. For the fifth round of selection, 0.06 μM xUba1(UFD), 5 μM xUbcH7, and 0.1 μM biotin–xUB were used, and the reaction time was shortened to 10 min.

#### Assaying the activity of the selected RBR mutants of Parkin

The RBR mutants from phage selection were cloned into the pGEX vector using primers GC6 and GC7 between restriction sites BamHI and NotI to express the proteins with an N-terminal GST tag. Ubiquitination of the RBR was set up with 5 μM RBR mutant, 0.1 μM xUba1, 2 μM xUbcH7, and 25 μM HA-xUB in buffer with 50 mM Tris-HCl (pH 7.5), 10 mM MgCl_2_, and 1.5 mM ATP. The reaction was incubated at 37°C for 10 min and was subject to SDS-PAGE and western blotting with an anti-Flag antibody to identify RBR mutants that could mediate the transfer of xUB. To incorporate mutations of RBR into the full-length *PRKN* gene, primers GC8 and GC7 were used to amplify the RBR gene. The fragment was assembled with the PCR fragment generated with the GC9/GC10 pair and cloned into the pGEX vector between restriction sites BamHI and NotI for expression of full-length Parkin with RBR mutations. To test the activity of Parkin for Miro1 ubiquitination, reactions were set with 20 μM wt UB, 1 μM wt Uba1, 10 μM wt UbcH7, 5 μM wt Parkin, 5 μM Miro1, and 10 μL 5× reaction buffer (250 μM DTT, 25 mM ATP, 25 mM MgCl_2_, pH 8.0 in PBS) in a total volume of 50 μL. The reaction tubes were shaken at 37°C overnight before analysis by SDS-PAGE and western blot.

#### Protein expression from recombinant pET and pGEX plasmids

Recombinant pET plasmids for the expression of Uba1, UbcH7, UB, and Rab1a, Rab5a, Rab5c, Rab7a, Rab8a, Rab10, Rab13, and CDK5 were transformed into BL21 cells and cultured in 2×YT broth with antibiotics under 37°C until the OD values of the cultures were in the range of 0.6–0.8. IPTG was added to the cell culture to reach the final concentration of 1 mM to induce protein expression, and the cell culture was incubated overnight under 20°C with agitation at 220 rounds per minute (rpm) before the cells were harvested by centrifugation (5,500 rpm, 4°C, 20 min). Cells were resuspended in 20 mL of lysis buffer (50 mM NaH_2_PO_4_, 300 mM NaCl, 10 mM imidazole, pH 8.0) with the addition of 40 mg lysozyme and 1 mM PMSF, and the suspension was incubated on ice for 30 min. The cells were lysed by sonication on ice, and the cell lysates were centrifuged (10,000 rpm, 4°C, 30 min). After centrifugation, the supernatant of the lysate was collected to bind with Ni-NTA beads overnight at 4°C. The protein was purified by a gravity-flow column with washes by 20 mL of lysis buffer (50 mM NaH_2_PO_4_, 300 mM NaCl, 5 mM imidazole, pH 8.0) once and 20 mL wash buffer (50 mM NaH_2_PO_4_, 300 mM NaCl, 20 mM imidazole, pH 8.0) twice followed by elution with 5 mL of elution buffer (50 mM NaH_2_PO_4_, 300 mM NaCl, 250 mM imidazole, pH 8.0). The eluted protein solution was further dialyzed against a dialysis buffer (50 mM Tris, 50 mM NaCl, 1 mM DTT, pH 8.0) and concentrated.

Recombinant pGEX plasmids for the expression of wt full-length Parkin and xParkin and the wt and mutant RBR domains were also transformed into BL21 cells, and the colonies acquired were used to inoculate 2×YT broth with ampicillin (100 μg/mL) and cultured at 37°C. 1 mM IPTG was added to the cell culture during log phase growth to induce protein expression, and the culture was incubated overnight at 20°C with agitation (220 rpm) before the cells were harvested by centrifugation (5,500 rpm, 4°C, 20 min). Cells were resuspended in 20 mL lysis buffer (50 mM Tris, 150 mM NaCl, pH 8.0, freshly added with 1 mg/mL lysozyme, 50 μM ZnCl_2_, 1 mM PMSF, and 10 mM DTT), and incubated on ice for at least 30 min. The cell suspension was sonicated for 30 min on ice, and the crude cell lysate was centrifuged at 14,000 rpm for 30 min to remove cell debris. The clear supernatant was collected into a clean 50 mL tube. 1 mL of GST beads was washed 3 times with 1 mL lysis buffer and then added into cell lysate. The mixture was put on a rotator at 4°C for overnight binding. The next morning, the mixture was applied to a clean affinity column and was washed sequentially by 15 mL lysis buffer, 2 × 15 mL wash buffer (50 mM Tris, 150 mM NaCl, pH 8.0), and eluted with 5 mL elution buffer (50 mM Tris, 150 mM NaCl, 10 mM reduced glutathione, pH 8.0). Eluate was sealed in a dialysis bag and then put into 1 L dialysis buffer (25 mM Tris base, 150 mM NaCl, 50 μM DTT, pH 8.0) for incubation with slow rotation at 4°C overnight. The third day, eluate was dialysis for another 4 h in 1 L of fresh dialysis buffer. Finally, the eluate was poured into a centrifugation concentrator with 5 kDa-cutoff and centrifuged at 4,000 rpm till the volume decreased to 0.5–1 mL. The final protein solution was aliquoted and stored at −80°C.

#### Tandem purification of HBT-xUB-conjugated proteins from cells expressing the Parkin OUT cascade

pLenti-xParkin and pLenti-xParkin with the inactivating C431A mutation were co-transfected with pLenti-HBT-xUB into the HEK293 cells with stable expression of xUba7-xUbcH7 pair^52^ to express the Parkin OUT cascade and the control OUT cascade in the cell. For transfection, xUba1-xUbcH7 cells were cultured in four 75 cm^2^-flasks and 5 μg of each of pLenti-HBT-xUB and pLenti-myc-xParkin or xParkin C431A plasmid were co-transfected into each flask of cells by DharmaFECT kb DNA transfection reagent (T-2006-01). After 48-h of transfection, cells were treated with 10 μM MG132 for 4 h at 37°C and washed twice with 2 mL ice-cold PBS. The buffer was then removed, and cells in each flask were lysed with 1 mL buffer A (8 M urea, 300mM NaCl, 50 mM Tris, 50 mM NaH_2_PO_4_, 0.5% NP-40, 1 mM PMSF and 125 U/ml benzonase, pH 8.0). The lysate was combined into a 15 mL Falcon tube and was sonicated for 2 min with a 5-s on and 5-s off cycle. After sonication, the lysate was centrifuged at 13,000 rpm for 30 min at room temperature. Clear supernatant was collected and total protein concentration was measured by the Bradford assay. Next, 35 μL PBS-washed Ni Sepharose High Performance beads (GE Healthcare, Cat# 17-5268-01) were added to each 1 mg of total protein (at least 10 mg in total), and a final concentration of 10 mM imidazole was added to the bead mixture for binding overnight on a rotator at room temperature. After binding, the Ni-NTA beads were loaded to a gravity column. Beads were washed sequentially with 20-bead volume of buffer A (pH 8.0), buffer A (pH 6.3), and buffer A (pH 6.3) with 10 mM imidazole, and eluted twice with 5-bead volume of buffer B (8 M Urea, 200 mM NaCl, 50 mM Na_2_HPO_4_, 2% SDS, 10 mM EDTA, 100 mM Tris, 25 mM imidazole, pH 4.3). The pH of the combined eluate was adjusted to 8.0, and 10 μL Pierce high-capacity streptavidin agarose resin (Thermo Scientific, Cat# 20359) was added to each 1 mg of protein in the beginning cell lysate for overnight binding at room temperature. The streptavidin beads were then washed sequentially with 1.5 mL of buffer C (8 M rea, 200 mM NaCl, 2% SDS, 100 mM Tris, pH 8.0), 1.5 mL buffer D (8 M Urea, 1.2 M NaCl, 0.2% SDS, 100 mM Tris, 10% EtOH, 10% Isopropanol, pH 8.0), and finally resuspend with 1.5 mL buffer E (8 M urea, 100 NH_4_HCO_3_, pH 8.0).

#### Sample digestion and LC-MS/MS analysis

Residual buffer E was removed from the mixture with the streptavidin resin from the previous step, and 200 μL of 50 mM NH_4_HCO_3_ was added to each sample, which were then reduced with dithiothreitol (final concentration 1 mM) for 30 min at 25°C. This was followed by 30 min of alkylation with 5 mM iodoacetamide in the dark. The samples were then digested with 1 μg of Lysyl-endopeptidase (Wako) at room temperature for 2 hours and further digested overnight with 1:50 (w/w) trypsin (Promega) at room temperature. The resulting peptides were acidified with 25 μL of 10% (v/v) formic acid (FA) and 1% (v/v) trifluoroacetic acid (TFA) and desalted with a Sep-Pak C18 column (Waters). For desalting, the Sep-Pak column was washed with 1 mL of methanol and 1 mL 50% (v/v) acetonitrile (ACN). The column was equilibrated by washing with 2 rounds of 1 mL of 0.1% (v/v) TFA in water. The acidified peptides were then loaded to the column, and the column was washed with 2 rounds of 1 mL 0.1% (v/v) TFA. Elution of the peptides from the column was carried out by 2 rounds of 50% (v/v) ACN (400 μL each). The peptide eluent was dried under a vacuum. Liquid chromatography coupled to tandem MS (LC-MS/MS) on an Orbitrap Fusion mass spectrometer (Thermo Fisher Scientific) was performed at the Emory Integrated Proteomics Core (EIPC) according to previously published methods.^52^ Collected spectra were searched using Proteome Discoverer 2.0 against the human UniProt database (90,300 target sequences). Searching parameters included full tryptic restriction and a parent ion mass tolerance (±20 parts per million). Methionine oxidation (+15.99492 Da), asparagine and glutamine deamidation (+0.98402 Da), lysine ubiquitination (+114.04293 Da), and protein N-terminal acetylation (+42.03670) were variable modifications (up to three were allowed per peptide); cysteine was assigned a fixed carbamidomethyl modification (+57.021465 Da). Percolator was used to filter the PSMs to a false discovery rate of 1%.

#### Bioinformatics analysis

To identify PARKIN substrates from the OUT screen, we generated a volcano plot by calculating the *p*-value based on the PSM ratios of the proteins purified from OUT cells compared to the control cells (Log2[PSM ratio OUT/control] > 1). Protein targets with *p* < 0.1 and a PSM ratio >2 were classified as potential Parkin substrates. The enriched targets were used as input to generate the list of gene ontology in the Biological Process category and disease ontology in the up_kw_disease category using DAVID (https://david.ncifcrf.gov/). A threshold of FDR <0.05 was selected as the significant terms, and the top 15 terms were picked to output the bar plot (ggplot2). Target networks were generated using STRING (https://string-db.org). All heat maps with normalized PSM values were generated using the R pheatmap packages.

#### *In vitro* ubiquitination of substrate proteins

Reconstituted ubiquitination reactions were set up in 50 μL of reaction buffer (50 mM Tris, 5 mM MgCl_2_, 5 mM ATP, and 50 μM DTT). Substrate proteins (10 μM) were incubated with 0.5 μM wt Uba1, 2 μM wt UbcH7, 10 μM wt HA-UB and 0.6 μM wt full-length human Parkin protein (R&D Systems) at 37°C for 4 h. A final concentration of 1 μM human PINK1 enzyme (R&D Systems) was also added to the reaction to activate Parkin. Parallel control reactions were set up, with each enzyme component excluded from the reaction mixture. The reactions were quenched by boiling the samples in the SDS-PAGE loading dye with 50 mM DTT for 5 min and analyzed by SDS-PAGE and western blot probed with the substrate-specific antibodies.

#### Cell-based assay to verify Parkin-catalyzed ubiquitination of the substrate proteins

HEK293T cells were transfected with varying amounts of pLenti-wtParkin plasmid (0–4 μg) for 14 h. The cells were then treated with 0.5 μM MG132 for an additional 12 h and washed twice with ice-cold PBS, pH 7.4, followed by the addition of 1 mL ice-cold RIPA buffer for incubating with the cells at 4°C for 10 min. The cells were lysed by repeated aspiration through a 21-gauge needle, and the cell lysate was transferred to a 1.5 mL tube. The cell debris in the lysate was pelleted by centrifugation at 13,000 rpm. for 20 min at 4°C, and the supernatant was transferred to a new tube and precleared by adding 1.0 μg of the appropriate control IgG (mouse or rabbit IgG corresponding to the host species of the primary antibody). 20 μL of suspended protein A/G PLUS-agarose was added to the supernatant, and the incubation was continued for 30 min at 4°C. After the prebinding step, cell lysate containing 2 mg total protein was transferred to a new tube, and 30 μL (i.e., 6 μg) primary antibody specific for the substrate proteins (Rab1a, Rab5a, Rab5c, Rab7a, Rab8a, Rab10, Rab13, and CDK5) were added to bind to target proteins in different batches of lysate. The incubation was continued for 2 hours at 4°C, and 30 μL of resuspended protein A/G PLUS-agarose was added. The tubes were capped and incubated at 4°C on a rocking platform overnight. The next day, the agarose beads were pelleted by centrifugation at 350*g* for 5 min at 4°C. The beads were then washed three times, each time with 1.0 mL PBS. After the final wash, the beads were resuspended in 40 μL of 1× Laemmli buffer with β-mercaptoethanol. The samples were boiled for 5 min and analyzed by SDS-PAGE. The western blots of the PAGE gels were probed with an anti-UB antibody.

#### Protein degradation assays

For revealing the effect of Parkin on the steady-state levels of substrates, HEK293T cells (5 × 10^6^ cells) were transiently transfected with varying amounts of pLenti plasmid of Parkin with the DharmaFECT kb transfection kit. Cells were harvested at 16 h after transfection, and the amount of substrate proteins in the cell lysate was assayed by immunoblotting with substrate-specific antibodies.

#### Substrate ubiquitination under mitophagy stimulation

Transfection of pLenti-Parkin into the HEK293T cells was conducted with the DharmaFECT transfection reagents according to the manufacturer’s protocol. After overnight transfection, the cells were treated with 0.5 μM MG132 for an additional 10–12 h. For assaying the effect of Parkin activation on the ubiquitination of the substrates during mitophagy, the cells were treated with 4 μM antimycin A and 10 μM oligomycin (AO) for 1 h, and substrates were immunoprecipitated with substrate-specific antibodies to assay their ubiquitination level. The immunoprecipitation of the Parkin substrates and assaying their ubiquitination levels with western blots followed the procedures in the previous sections.

#### Cancer proteomic and genomic analyses

The protein-protein and RNA-RNA correlation data of Breast Cancer (BRCA) was downloaded from LinkedOmicsKB (https://kb.linkedomics.org/download, data obtained on May 28th, 2024). The protein levels and RNA levels of PRKN (ENSG00000185345.23), RAB1A (ENSG00000138069.18) and RAB7A (ENSG00000075785.13) were extracted from LinkedOmicsKB files 'BRCA_proteomics_gene_abundance_log2_reference_intensity_normalized_Tumor.txt' and 'BRCA_RNAseq_gene_RSEM_coding_UQ_1500_log2_Tumor.txt', respectively. For correlation analysis, data on protein levels and RNA levels were merged on patient ID, and 88 samples that were aligned with both proteomic and genomic data were selected for the statistical analysis. Spearman correlation coefficient (rho) and associated *p*-values were calculated with SciPy package written in Python. Scatterplots and linear regression were drawn with Seaborn package written in Python.

### Quantification and statistical analysis

Representative results of the ubiquitination assays were shown in the figures based on two to three independent experimental repeats. For identifying Parkin substrates using the OUT cascade, three independent purifications were carried out (*N* = 3 independent biological replicates). Proteins with Log_2_[PSM ratio OUT/control] >1 and -Log_10_*p* > 1 were identified as potential Parkin substrates. A paired T-test was performed to calculate *p* values based on the PSM ratios of the proteins purified from OUT cells expressing the Parkin OUT cascade compared to the control cells expressing an inactive xPARKIN C431A mutant. The volcano plot was generated by ggplot2 (Figures 4C and S5B). Functional annotation pathway analysis was performed using DAVID (https://david.ncifcrf.gov/). A threshold of FDR <0.05 was selected as the significant term, and the top 15 terms were picked to generate the bar plots using ggplot2 (Figures 5A and S7B). Target networks were generated using STRING (https://string-db.org) (Figures 4D and S7). All heat maps with normalized PSM values were generated using the R pheatmap packages (Figures 5C, S6, and S7A).

## Supplemental information

Document S1. Figures S1–S8

Table S1. Data for the proteomics screen of Parkin substrates

Table S2. Assignment of potential substrates of Parkin based on the calculation of the Log2[PSM ratio OUT/Control cells] and -Log10P values

Table S3. Overlaps of the Parkin substrate profile between the OUT screen and other reported studies
