# Supplementary material for: Engineering a cell-based orthogonal ubiquitin transfer cascade for profiling the substrates of RBR E3 Parkin
Source: iScience. 2025 Jun 17;28(7):112913. doi: 10.1016/j.isci.2025.112913 (PMC12271601; doi:10.1016/j.isci.2025.112913)
Supplement: Document S1. — Figures S1–S8 [file mmc1.pdf]

iScience, Volume *28*

Supplemental information

Engineering a cell-based orthogonal ubiquitin transfer cascade for profiling the substrates of RBR E3 Parkin

Shuai Fang, Li Zhou, Geng Chen, Jing Zhang, Xiaoyu Wang, In Ho Jeong, Savannah E. Jacobs, Bradley R. Kossmann, Wei Wei, Shu Liu, Geon H. Jeong, Yayun Xie, Duc Duong, Nicholas T. Seyfried, Ivaylo Ivanov, Angela M. Mabb, Hiroaki Kiyokawa, Bo Zhao, and Jun Yin

Supplemental Figures

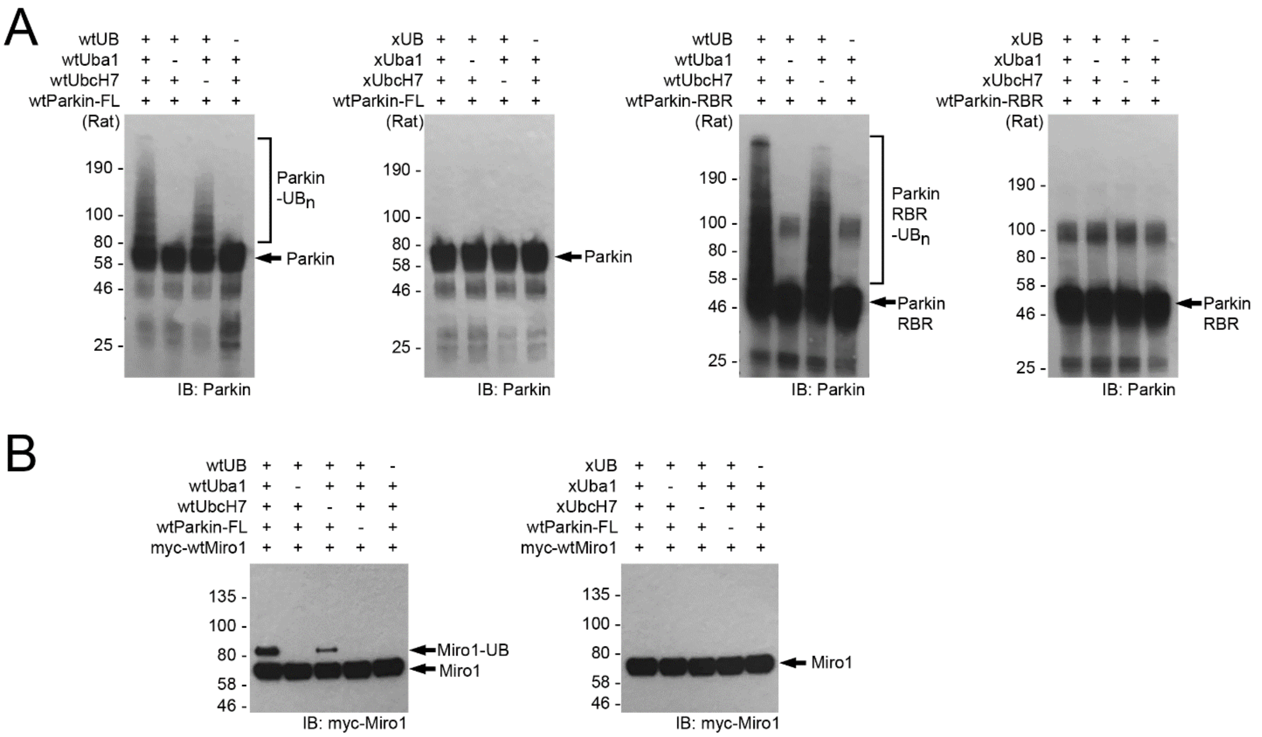

Figure S1. wt rat Parkin was not active in xUB transfer in the self-ubiquitination reaction and the ubiquitination of Miro1. (A) Full-length rat Parkin and the RBR domain were active in pairing with wt Uba1 (E1) and UbcH7 (E2) for utilizing wt UB for the self-ubiquitination reaction but were incapable of transferring xUB with the xUba1-xUbcH7 pair. Parkin and its RBR domain were expressed as GST fusions. (B) Full-length rat Parkin could transfer wt UB to the Parkin substrate Miro1 but was incapable of transferring xUB to Miro1 with the xUba1-xUbcH7 pair.

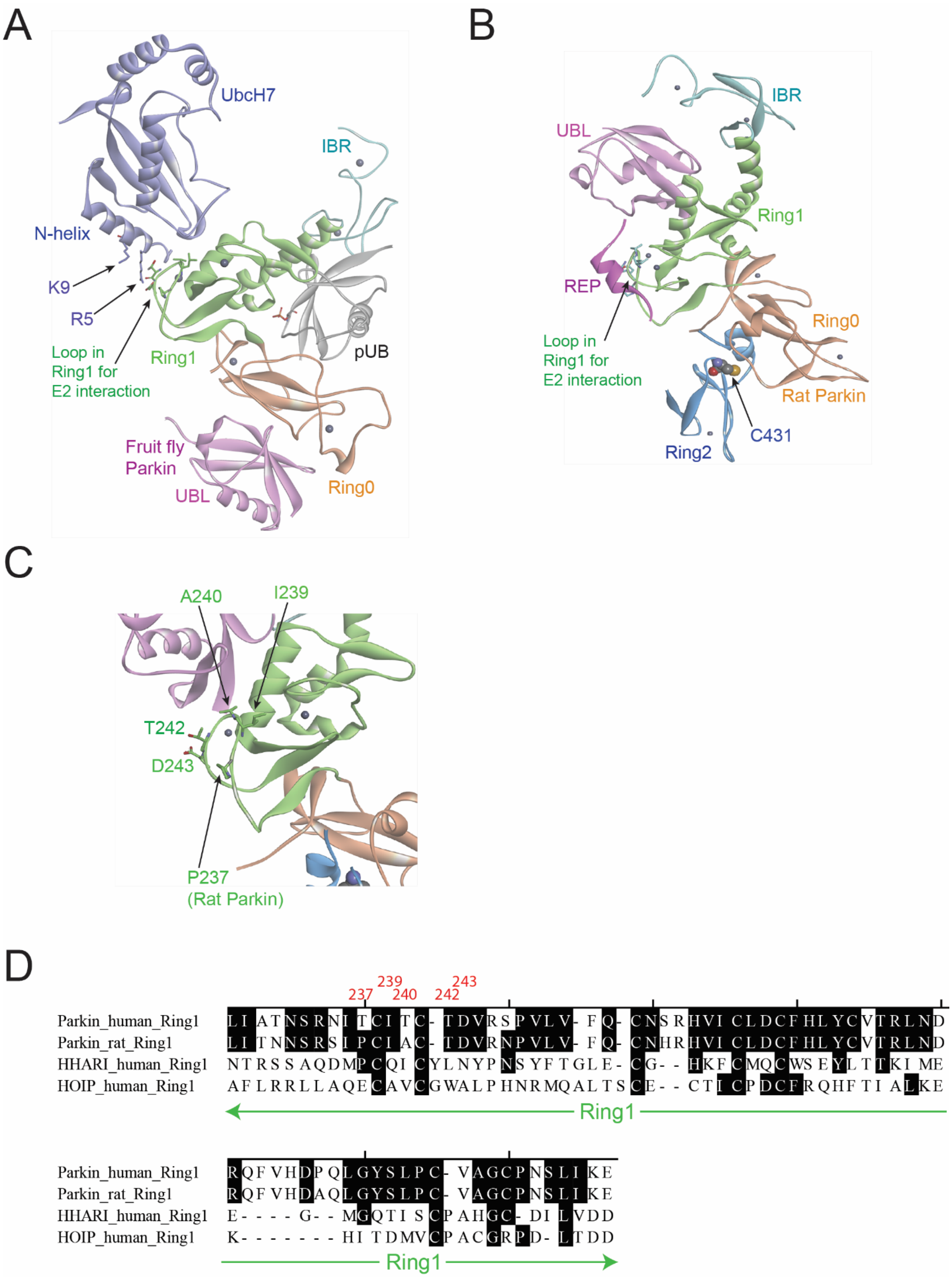
A
UbcH7
IBR
N-helix
K9
R5
Loop in
Ring1 for
E2 interaction
Ring1
pUB
Fruit fly
Parkin
UBL
Ring0
B
IBR
UBL
Ring1
REP
Loop in
Ring1 for
E2 interaction
Ring0
Rat Parkin
C431
Ring2
C
A240
I239
T242
D243
P237
(Rat Parkin)
D
237 239 240 242 243
Parkin_human_Ring1 LIATNSRNITCITC-TDVRSPVLV-FQ-CNSRHVICLDCFHLYCVTRLND
Parkin_rat_Ring1 LITNNSRSIPCIAC-TDVRNPVLV-FQ-CNHRHVICLDCFHLYCVTRLND
HHARI_human_Ring1 NTRSSAQDMPCQICYLNYPNSYFTGLE-CG--HKFCMQCWSEYLTTKIME
HOIP_human_Ring1 AFLRRLLAQECAVCGWALPHNRMQALTSCE--CTICPDCFRQHFTIALKE
Ring1
Parkin_human_Ring1 RQFVHDPQLGYSLPC-VAGCPNSLIKE
Parkin_rat_Ring1 RQFVHDAQLGYSLPC-VAGCPNSLIKE
HHARI_human_Ring1 E----G--MGQTISCPAHGC-DILVDD
HOIP_human_Ring1 K-------HITDMVCPACGRPD-LTDD
Ring1

Figure S2. UbcH7-Parkin interaction between the N-terminal helix of UbcH7 and the loop region of the Ring1 domain of Parkin. (A) The crystal structure of Parkin from *B. dorsalis*, an oriental fruit fly, with UbcH7 bound to the Ring1 domain of Parkin (PDB ID: 6DJX).[61] (B) Crystal structure of rat Parkin showing essential domains of the RBR E3 with the REP element shielding the loop region of the Ring1 domain and blocking the binding of E2 such as UbcH7 to Parkin (PDB ID: 4K95).[63] (C) A detailed view of the loop residues in the Ring1 domain of rat Parkin that may bind to the N-terminal helix of UbcH7. The highlighted residues in the loop of Ring1, including P237, I239, A240, T242 and D243, may bind to R5 and K9 residues in the N-terminal helix of UbcH7. (D) The alignment of the protein sequences of the Ring1 domain of RBR E3s Parkin, HHARI and HOIP. Residue numbering follows the human Parkin sequence.

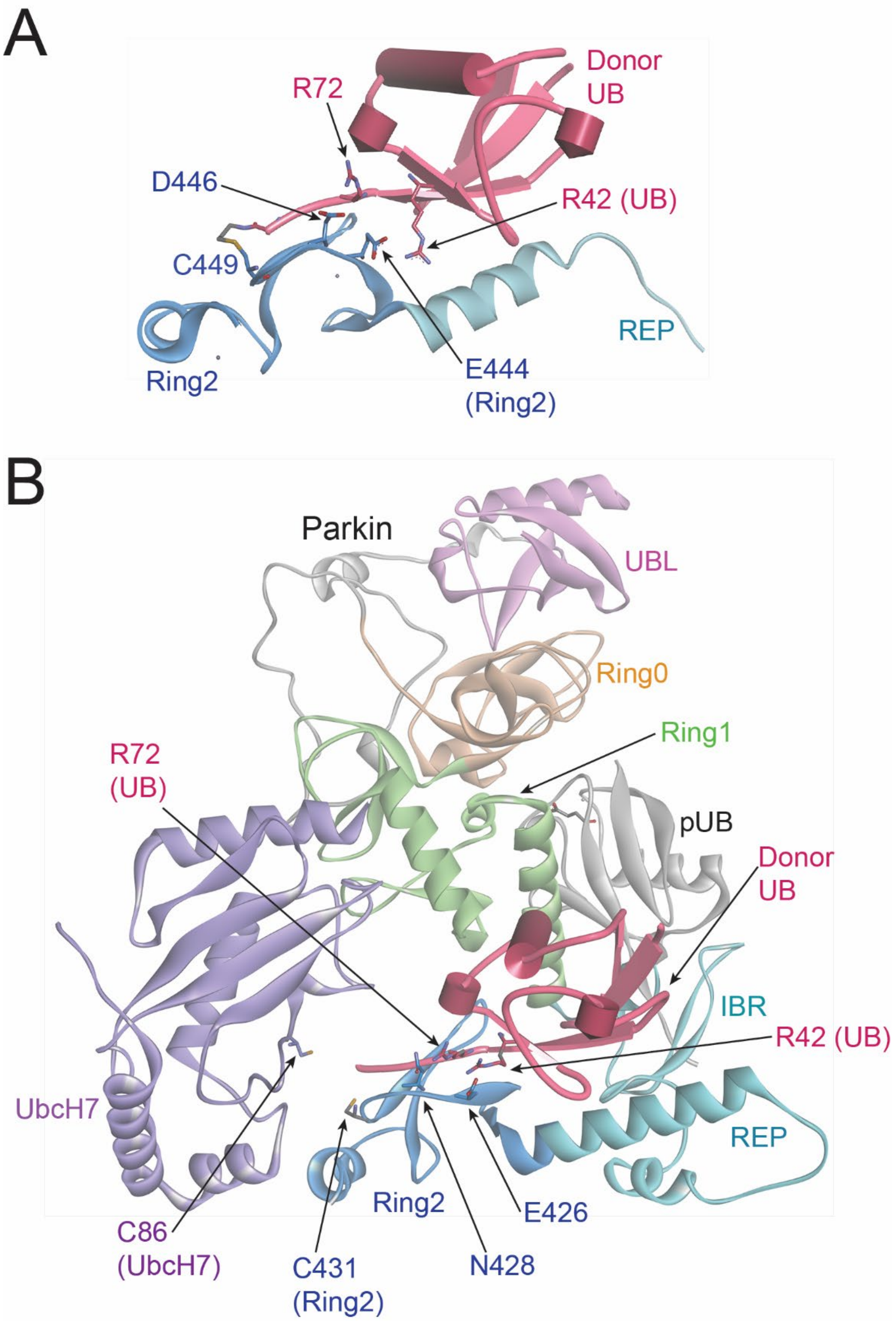

Figure S3. Interaction of the donor UB with the Ring2 domain of Parkin during the transthiolation reaction. (A) Structure of the Ring2 domain of *D melanogaster* Parkin in a covalent conjugate with the donor UB through the catalytic C449 (PDB ID 9C5E).[65] E444 and D446 in the Ring2 domain, equivalent

to E426 and N428 in the Ring2 domain of human Parkin, are in proximity with R42 and R72 residues in the donor UB that were mutated to Glu residues in xUB. (B) Modelled structure of human Parkin in the catalytic active state undergoing the transthiolation reaction for transferring the donor UB from UbcH7 to C431 of Parkin.[65] E426 and N428 in the Ring2 domain of Parkin interact with R42 and R72 of the donor UB to facilitate UB transfer.

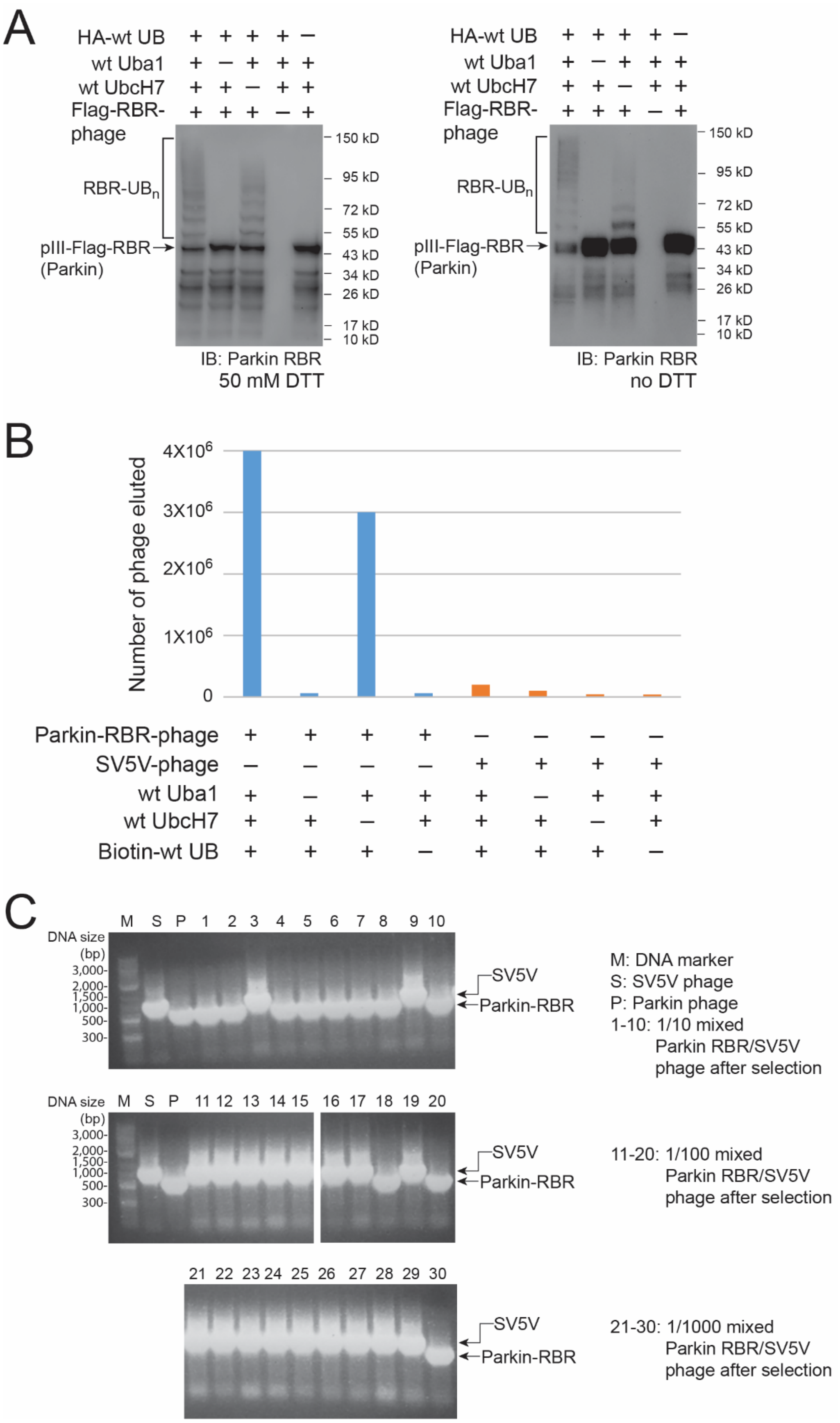
A
HA-wt UB
wt Uba1
wt UbcH7
Flag-RBR-phage
RBR-UBn
pIII-Flag-RBR (Parkin)
150 kD
95 kD
72 kD
55 kD
43 kD
34 kD
26 kD
17 kD
10 kD
IB: Parkin RBR
50 mM DTT
IB: Parkin RBR
no DTT
B
Number of phage eluted
4X10^6
3X10^6
2X10^6
1X10^6
0
Parkin-RBR-phage
SV5V-phage
wt Uba1
wt UbcH7
Biotin-wt UB
C
DNA size (bp)
3,000
2,000
1,500
1,000
500
300
SV5V
Parkin-RBR
M: DNA marker
S: SV5V phage
P: Parkin phage
1-10: 1/10 mixed Parkin RBR/SV5V phage after selection
11-20: 1/100 mixed Parkin RBR/SV5V phage after selection
21-30: 1/1000 mixed Parkin RBR/SV5V phage after selection

Figure S4. Western blot analysis of rat Parkin RBR-UB conjugates formed on phage surface and model selection of phage displaying the wt Parkin RBR domain. (A) Western blots of the self-ubiquitination reactions of the rat Parkin-RBR domain displayed on the phage surface. The ubiquitination reactions were boiled in the gel loading dye with 50 mM dithiothreitol (DTT) (left panel) or without DTT (right panel) before analysis by SDS-PAGE. The western blots of the gel were probed with an anti-Parkin antibody that recognized the Parkin RBR domain. (B) Phage displaying the wt RBR domain of rat Parkin were reacted with biotin-wt UB and the wt Uba1-UbcH7 pair. In parallel, control reactions were set up with the exclusion of either wt Uba1, UbcH7 or biotin-wt UB from the reaction mixture. In another set of controls, phage displaying a viral protein SV5V with no UB ligase activity were reacted with biotin-wt UB and the wt Uba1-UbcH7 pair. After the reactions, phage particles were bound to the streptavidin plate to retain phage conjugated with biotin-wt UB. The streptavidin plate was then washed to remove unreacted phage, and the phage particles bound to the plate were eluted by the addition of an elution buffer containing 10 mM DTT. The eluted phage particles were titered, and their numbers were plotted in the chart. (C) Colony PCR reactions to identify phage displaying the wt RBR domain of Parkin after model selection with a 1/10, 1/100 and 1/1,000 mixture of RBR and SV5V displayed phages. PCR amplification of the gene encoding the RBR domain of Parkin in the pComb vector in the *E. coli* colonies gave a fragment size of 750 bp compared to the amplification of the SV5V fragment with a size of 900 bp.

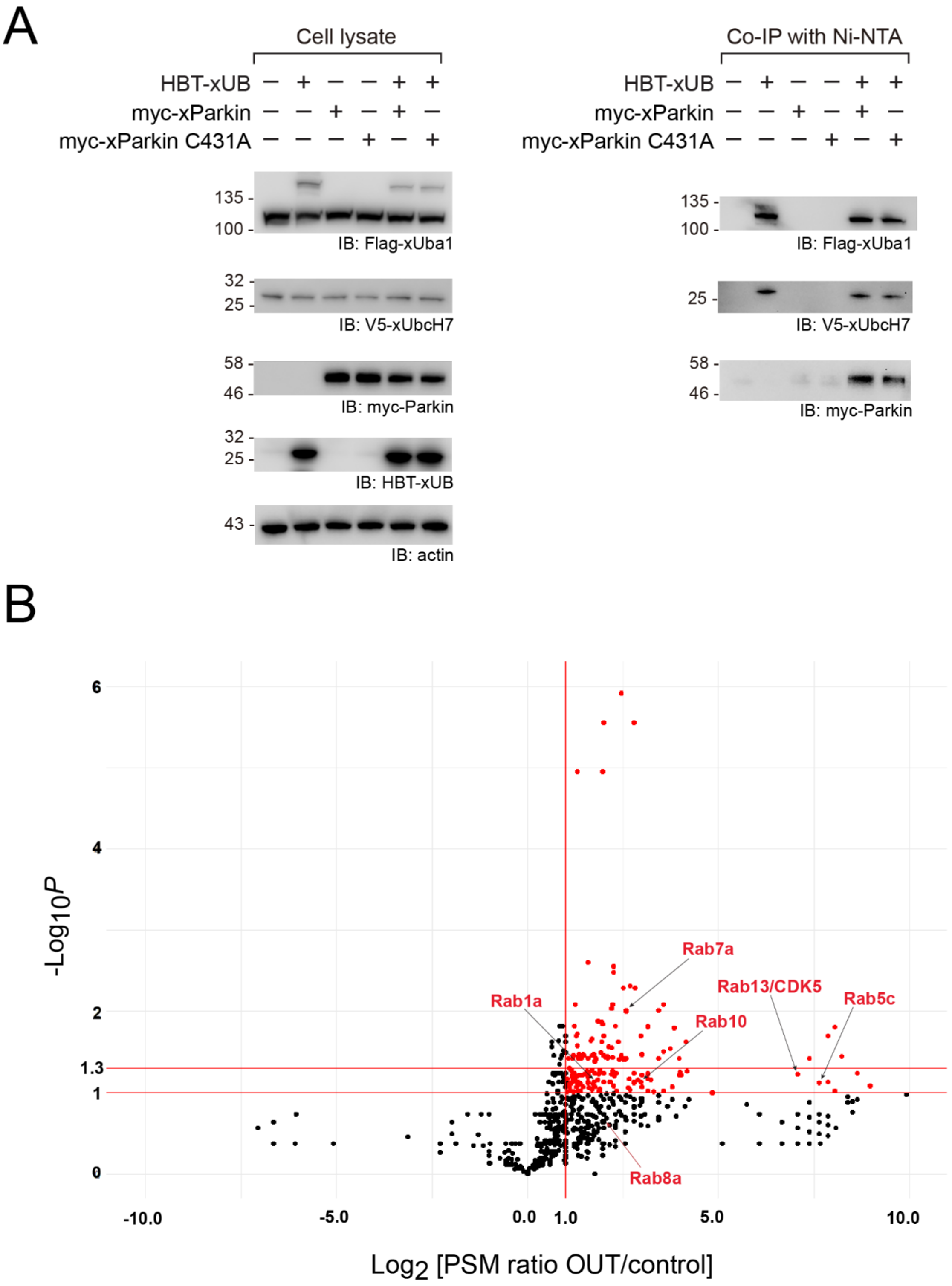

Figure S5. Expression of the Parkin OUT cascade in HEK293 cells and the volcano plot of Parkin substrates identified by the OUT screen. (A) HEK293 cells stably expressing the xUba1-xUbcH7 pair

were transfected with the individual plasmids for expressing HBT-xUB, xParkin, or xParkin C431A mutant or a combination of the plasmids as designated. The expression of the OUT cascade components in the cell was shown in the western blots on the left, and the purification of the OUT components as HBT-xUB conjugated proteins bound to the Ni-NTA resin was shown on the right. HBT-xUB was probed with a streptavidin-horseradish peroxidase (HRP) conjugate. (B) Volcano plots of the proteins conjugated with HBT-xUB from three repeats of tandem purification from the OUT and control cells. N = 3 independent biological replicates. Red dots designate proteins with Log_2[PSM ratio OUT/control] >1 and $-Log_{10}P > 1$.

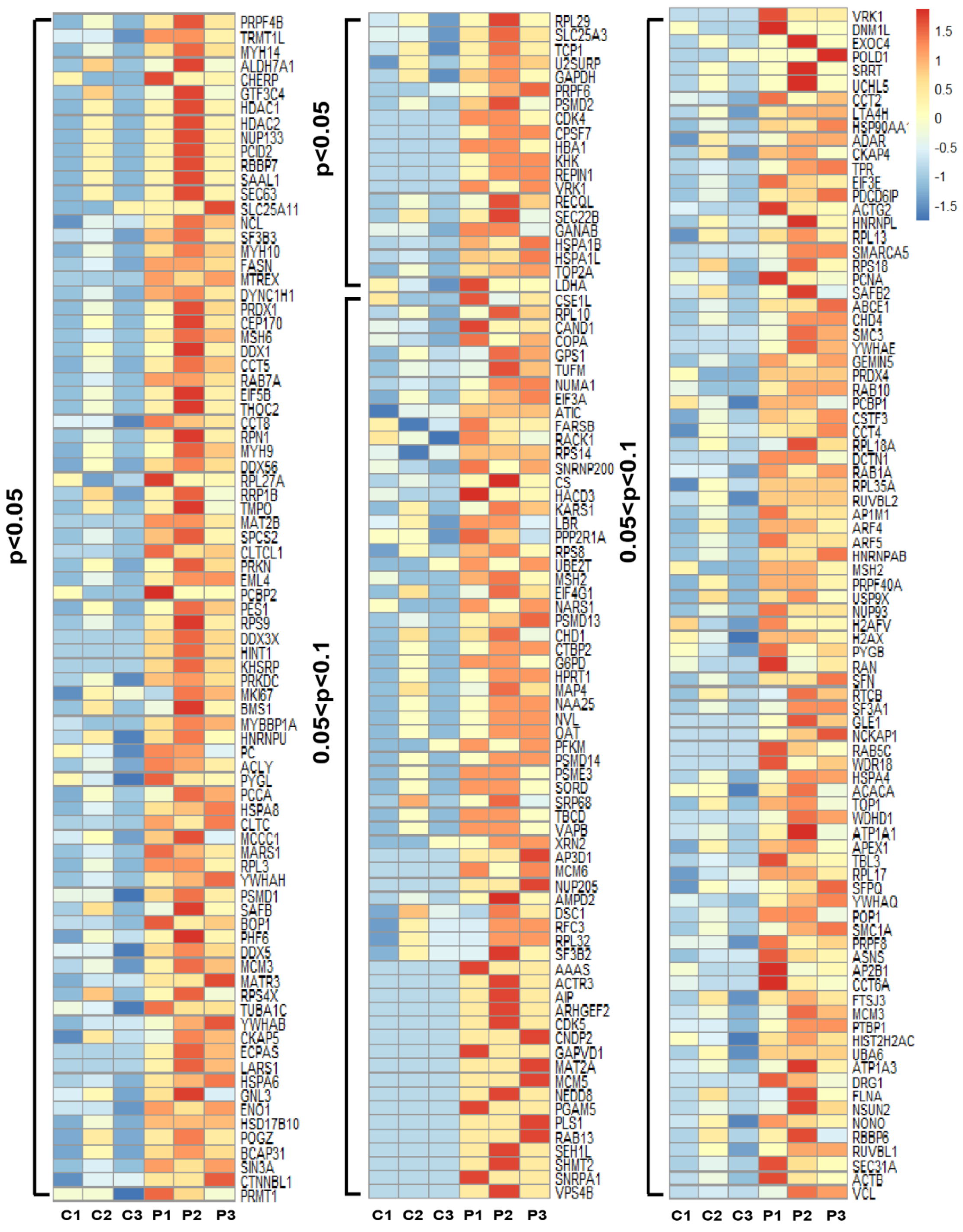

Figure S6. Heat map with normalized PSM values for the proteins identified as potential Parkin substrates by the OUT screen. The ranges of the p values are shown in the graph. P1-P3: 3 replicates from OUT cells expressing the functional OUT cascade of Parkin; C1-C3: 3 replicates from the control cells expressing the C431A mutant of xParkin.

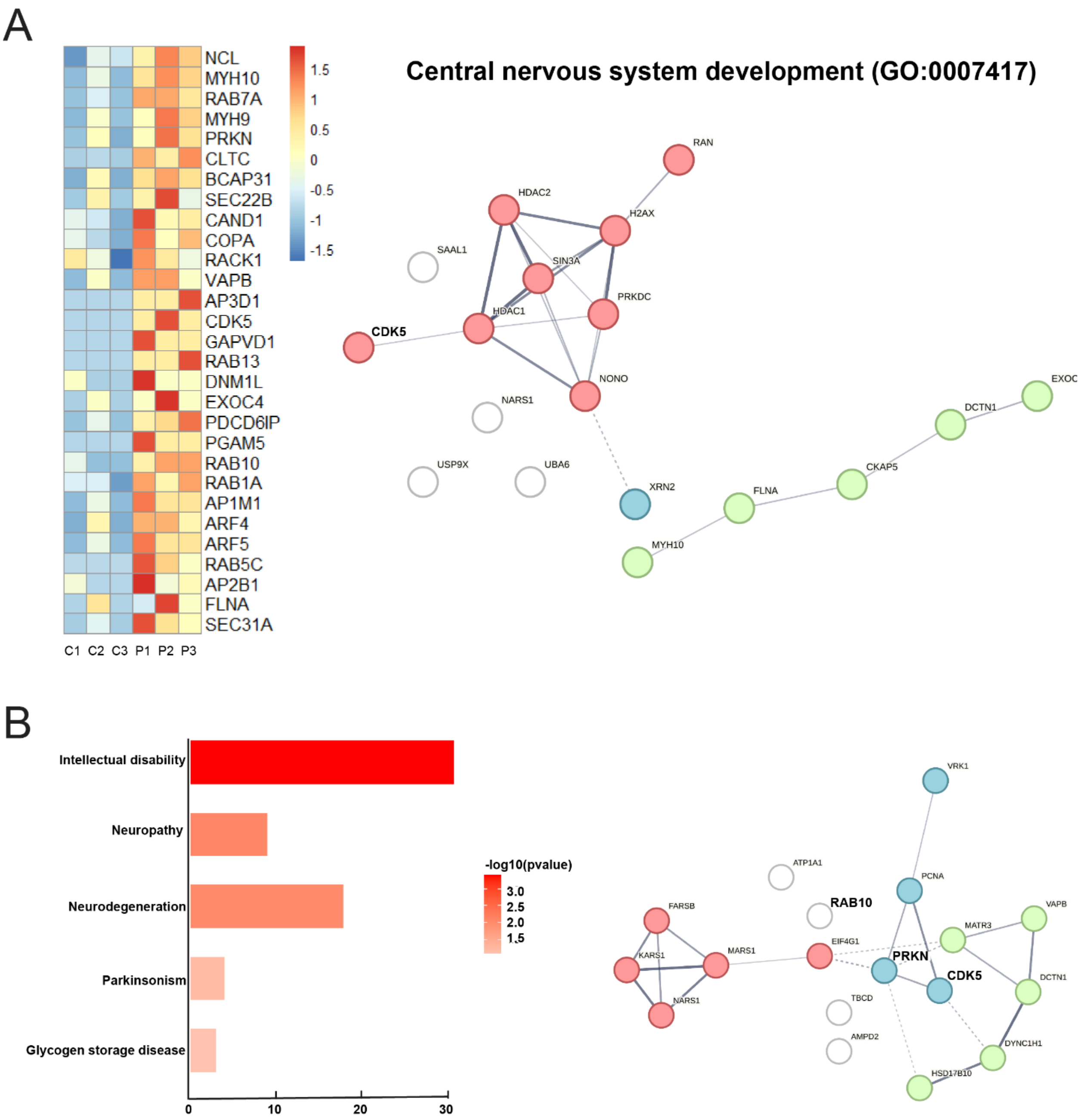

Figure S7. Analysis of Parkin substrates associated with neurodevelopment and diseases. (A) *Left,* Heatmap with normalized PSM values for the targets identified from OUT that were assigned to central nervous system development. *Right,* STRING protein-protein interaction network for targets identified from OUT that were assigned to central nervous system development (GO:0007417). (B) *Left*, Visualization of top significant terms from the list of disease-associated annotations from DAVID based

on the analysis of Parkin substrates from the OUT screen. *Right,* STRING protein-protein interaction network of the Parkin substrates involved in neurodegeneration (KW-0523).

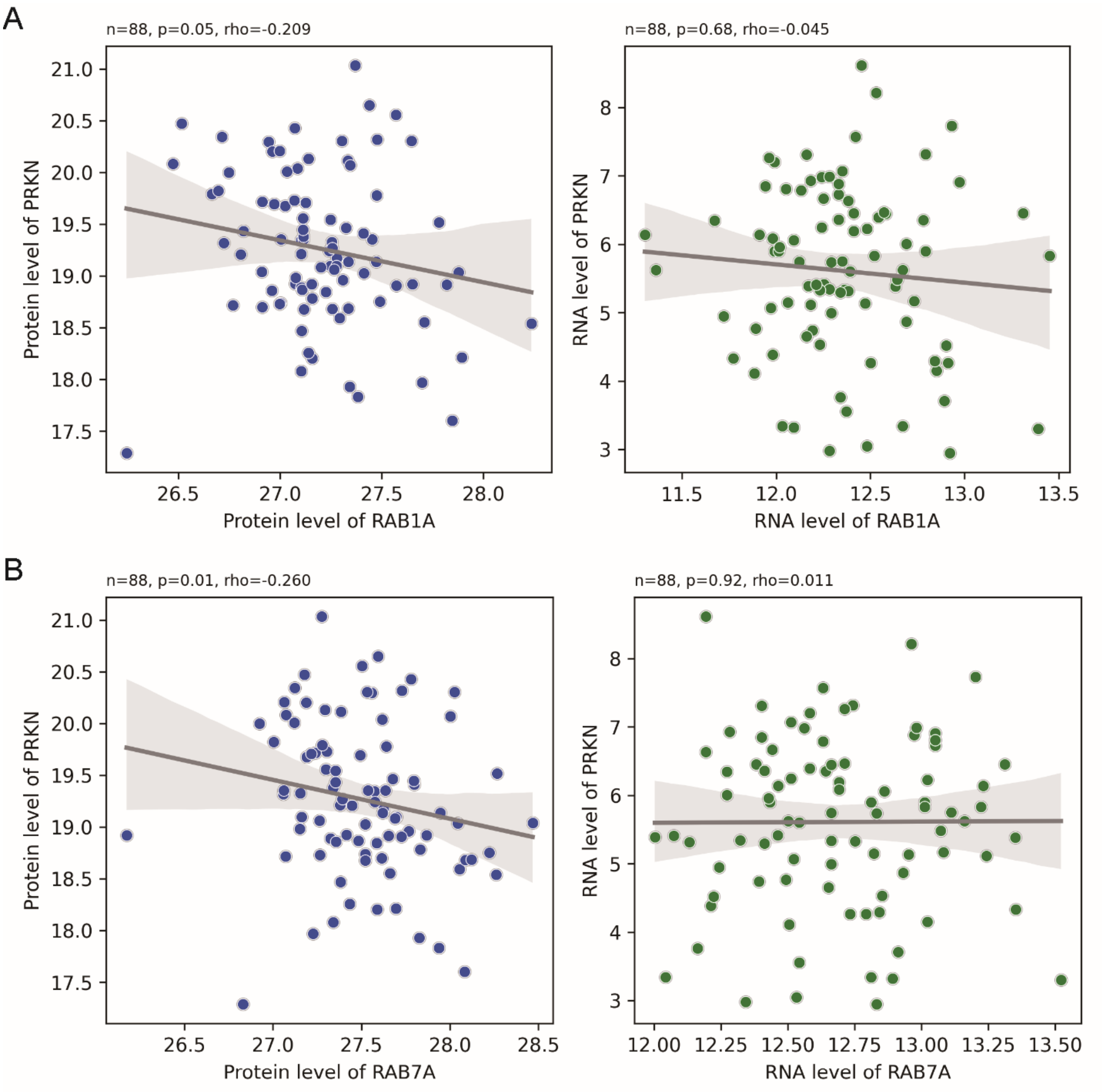

Figure S8. The protein level of Parkin is reciprocally correlated with those of Rab1a and Rab7a in human breast cancer tissues, whereas there are no significant correlations between the mRNA levels of Parkin and those of the Rab proteins. Data from each sample of breast cancer tissues were obtained from the LinkedOmicsKB proteogenomics database (https://kb.linkedomics.org/) and analyzed by Spearman correlation coefficient (rho) with associated p-values calculated with SciPy package written in Python.
